# CLPP Depletion Causes Diplotene Arrest; Underlying Testis Mitochondrial Dysfunction Occurs with Accumulation of Perrault Proteins ERAL1, PEO1, and HARS2

**DOI:** 10.3390/cells12010052

**Published:** 2022-12-22

**Authors:** Jana Key, Suzana Gispert, Lieke Koornneef, Esther Sleddens-Linkels, Aneesha Kohli, Sylvia Torres-Odio, Gabriele Koepf, Shady Amr, Marina Reichlmeir, Patrick N. Harter, Andrew Phillip West, Christian Münch, Willy M. Baarends, Georg Auburger

**Affiliations:** 1 Experimental Neurology, Medical Faculty, Goethe University, 60590 Frankfurt am Main, Germany; 2 Department of Developmental Biology, Erasmus Medical Center, 3015 GD Rotterdam, The Netherlands; 3 Oncode Institute, Erasmus Medical Center, 3015 GD Rotterdam, The Netherlands; 4 Institute of Biochemistry II, Goethe University Medical School, 60590 Frankfurt am Main, Germany; 5 Department of Microbial Pathogenesis and Immunology, College of Medicine, Health Science Center, Texas A&M University, Bryan, TX 77807, USA; 6 Institute of Neurology (Edinger-Institute), University Hospital Frankfurt, Goethe University, Heinrich-Hoffmann-Strasse 7, 60528 Frankfurt am Main, Germany; 7 Frankfurt Cancer Institute, 60590 Frankfurt am Main, Germany; 8 Cardio-Pulmonary Institute, 35392 Gießen, Germany

**Keywords:** meiosis-I, pachytene–diplotene, homologous recombination, H3K9ac, acetyl–tubulin, acSMC3, Twinkle helicase, RMND1, tRNA/rRNA processing, cGAS-STING signaling

## Abstract

Human Perrault syndrome (PRLTS) is autosomal, recessively inherited, and characterized by ovarian insufficiency with hearing loss. Among the genetic causes are mutations of matrix peptidase CLPP, which trigger additional azoospermia. Here, we analyzed the impact of CLPP deficiency on male mouse meiosis stages. Histology, immunocytology, different OMICS and biochemical approaches, and RT-qPCR were employed in CLPP-null mouse testis. Meiotic chromosome pairing and synapsis proceeded normally. However, the foci number of the crossover marker MLH1 was slightly reduced, and foci persisted in diplotene, most likely due to premature desynapsis, associated with an accumulation of the DNA damage marker γH2AX. No meiotic M-phase cells were detected. Proteome profiles identified strong deficits of proteins involved in male meiotic prophase (HSPA2, SHCBP1L, DMRT7, and HSF5), versus an accumulation of AURKAIP1. Histone H3 cleavage, mtDNA extrusion, and cGAMP increase suggested innate immunity activation. However, the deletion of downstream STING/IFNAR failed to alleviate pathology. As markers of underlying mitochondrial pathology, we observed an accumulation of PRLTS proteins ERAL1, PEO1, and HARS2. We propose that the loss of CLPP leads to the extrusion of mitochondrial nucleotide-binding proteins to cytosol and nucleus, affecting late meiotic prophase progression, and causing cell death prior to M-phase entry. This phenotype is more severe than in mito-mice or mutator-mice.

## 1. Introduction

Non-obstructive azoospermia (NOS, the complete absence of sperm from the ejaculate, due to a failure in spermatogenesis, leading to complete infertility) was observed so far in one of the few male individuals with a biallelic missense mutation in the gene encoding the mitochondrial matrix peptidase CLPP, and in all mice with a genetic depletion of this protein [1,2,3]. Its occurrence in CLPP mouse mutants represents an enigma because most previously known mitochondrial pathologies have much less impact on testis functions. Mitochondrial dysfunction usually only reduces sperm capacity and fertility to a degree known as asthenozoospermia [4].

In addition, human CLPP mutations result in primary ovarian insufficiency with early onset hearing loss, a feature combination known as autosomal recessive Perrault syndrome (PRLTS). Thus, CLPP depletion blocks germline differentiation completely, but there is very little knowledge about the molecular events that underlie this clinical condition [5,6,7]. CLPP-null mice appear to complete cell mitosis normally in most tissues, although their marked growth deficit does indicate a reduced cell division and/or increased cell death [8], but meiosis appears to be arrested selectively and completely.

Thus, our study asks the question of how a polypeptide cleavage impairment in mitochondria may be functionally connected to the progression of spermatogenesis, in particular meiotic prophase. In this survey, we aim to define the cytological features and prominent molecular anomalies and test their relevance for a therapeutic rescue of infertility.

PRLTS can also be caused by mutations in genes encoding other factors with functions at the mitochondrial nucleoid, tRNA/rRNA processing, and the mRNA translation apparatus, such as PEO1 (Twinkle), RMND1 (Required for Meiotic Nuclear Division 1 Homolog), PRORP, HARS2, LARS2, and ERAL1 [9,10,11]. While ERAL1 abundance has been shown to increase upon CLPP depletion, it remains unclear how the pathway connections are between CLPP and the other PRLTS disease proteins.

CLPP is phylogenetically conserved from bacteria such as *Escherichia coli* to plants such as *Arabidopsis thaliana* and also to mammals. It localizes to the matrix of bacteria, chloroplasts, or mitochondria, where bulk protein degradation is normally performed by the disaggregase and protease LONP1, while the activity of CLPP serves mostly in stress periods to compensate for protein misfolding and aggregation. Selective substrates that undergo CLPP-mediated decay (rather than LONP1-mediated decay) remain to be discovered, despite the vast literature in many organisms on proteome profiles and trap-assays of CLPP mutants [12].

CLPP monomeric peptidase subunits assemble into two heptameric rings, which interact on either side with hexameric rings of the AAA + ATPase CLPX as substrate-selecting disaggregase, forming a barrel-like structure. The chymotrypsin-like peptide-cleaving activity of CLPP is enhanced by CLPX to a degree wherein entire proteins can be degraded [13,14]. Upon CLPP depletion, an excessive accumulation of CLPX protein ensues [1]. There is also a co-accumulation with interactor proteins such as GRSF1, POLDIP2, LRPPRC, GFM1, and other factors known for their action at mitochondrial nucleoids, RNA granules, and mitoribosomes [15]. These are the most consistent consequences of CLPP depletion across different tissues in mice and humans, and they are accompanied by an enlargement of the nucleoid area and an increased dosage of mitochondrial DNA (mtDNA) [15]. It is important to note that, at least in mouse embryonic fibroblasts (MEFs), the excess CLPX/GRSF1/GFM1, as well as DNAJA3/STAT1 proteins redistribute to the cell nucleus, with parallel activation of widespread innate immune defenses against toxic DNA/RNA mediated by nuclear cGAS, diffusible cGAMP, and cytosolic STING [15,16,17,18]. Retrograde signaling of mitochondrial problems has long been known to trigger nuclear transcriptional responses [19,20] and may also perturb chromosomal replication and recombination.

Since meiosis-I in ovaries is initiated before birth, and the postnatal maturation of oocytes occurs only for a few cells in each menstrual cycle, it is quite demanding to define the molecular obstacles that cause primary ovarian insufficiency. Therefore, we focused on a testis which produces about 12 million sperm cells already in the first wave of spermatogenesis during the period between mouse postnatal days 10 and 35 (P10-P35) [21]. In later mouse life, spermatogenesis is continuously initiated but more asynchronized compared to the first wave. When the CLPP-null mouse was characterized in the past [1], analyses of cross-sections of seminiferous tubules revealed normal morphology and numbers for blood vessels, interstitial tissue, Leydig cells, and Sertoli cells, but germ cell development was severely affected, with a complete absence of post-meiotic cells. Here, we performed a detailed analysis of meiotic prophase progression, using immune histology and cytology. Important hallmarks of meiotic prophase progression are the induction of meiotic DNA double-strand breaks (DSBs) in a stage defined as leptotene, when the chromatin loops also start to become organized along a chromosomal axis that connects the loop bases by specialized proteins such as SYCP3 [22] that form part of these axial elements. Subsequently, meiotic DSB repair and chromosome pairing go hand in hand during zygotene, culminating in the formation of the synaptonemal complex (SC) between the now synapsed homologous chromosomes in pachytene. During this stage, a small percentage (around 10%) of the meiotic DSBs is repaired as crossover, and these sites are marked by the protein MLH1 [23]. Finally, in diplotene, the SC is disassembled (desynapsis) and cells then enter into metaphase-I, followed by the rapid completion of the first and subsequently also the second meiotic division. A microscopic analysis of meiotic prophase progression was then used to define at which stage a meiotic arrest might occur.

In advanced adult ages, a testicular inflammation and atrophy of CLPP-null testes may hamper analyses [1,24]. Therefore, three age points were selected for the proteome profiling of dysregulated molecules and enriched pathways in pathogenesis: P17 when meiosis-I should be near completion in primary spermatocytes, P21 when meiosis-II should just be finished in secondary spermatocytes, and P27 when round spermatids should start to elongate and acrosome/tail formation should be ongoing [25]. In addition, candidate mitochondrial CLPP degradation substrate proteins with significant excess abundance were assessed for their relocalization to the nucleus, and immunoblots with RT-qPCR were performed for validation and nuclear expression analysis.

These experiments allowed us to discriminate between primary changes in expression and those that are caused by the loss of specific cell types. Thus, the earlier dysregulations may be related to pathway disruptions and molecular deficiencies in the nucleus and cytosol, coupled with gain-of-function events for specific mitochondrial proteins. As a prime example, absent CLPP-mediated degradation leads to pathological accumulation of at least three other PRLTS disease proteins (namely ERAL1, PEO1/TWNK, and HARS2) in mitochondria, and increased mtDNA in the cytosol. HARS2 also accumulated in the nucleus.

Overall, our characterization of the CLPP-null mouse testis by microscopy, proteomics, RT-qPCR, and immunoblots provided (1) the crucial insight that spermatogenesis arrests before metaphase-I, (2) negative evidence on the relevance of inflammation for fertility, and (3) positive evidence that CLPP modulates key DNA/RNA-processing factors in mitochondria and the nucleus.

## 2. Materials and Methods

### 2.1. Mice and Mouse Embryonic Fibroblasts

The generation of the CLPP-null (*Clpp*^−/−^) mice was described in detail before [1], and pups were bred from heterozygous matings. Mice were housed under specific-pathogen-free conditions under a 12 h light cycle with food and water *ad libitum* in the central animal facility (ZFE) of the University Hospital Frankfurt. All animal experiments were performed in compliance with the German animal welfare law. Due to the complete infertility of CLPP-null homozygous mice of both sexes, breeding was performed simultaneously among multiple pairs of heterozygous mutation carriers, to then select male homozygous wild-type (WT) and mutant (null) offspring of matched birth date, aging them under identical conditions until sacrificed for analysis. CLPP-null mice were crossed with STING- and IFNAR- deleted mice as previously described [16].

Mouse embryonic fibroblasts (MEFs) were generated as previously reported [1] and cultivated in Dulbecco’s minimal essential medium (DMEM) 4.5 g/L glucose (Thermo Fisher Scientific, Waltham, MA, USA, #21969) supplemented with 15% fetal bovine growth serum (Gibco, Thermo Fisher Scientific), 1% Penicillin/Streptomycin (Gibco), and 1% Glutamine (Invitrogen, Waltham, MA, USA) at 37 °C and 5% CO_2_ in a humidified incubator, passaging every 3–4 days.

### 2.2. Antibodies

Anti-SYCP3 (1:5000, [26]), previously generated rabbit anti-HORMAD1 (1:200, [27]), previously generated rabbit anti-RPA (1:50, [28]), rabbit polyclonal anti-H3S10p (1:1000, Upstate, 06-570), mouse anti-MLH1 (1:25, BD Pharmingen, 51-1327GR), mouse monoclonal anti-γH2AX (1:10,000, Upstate, 05-636), and mouse monoclonal anti-SYCP3 (1:200, Abcam, ab97672) were used. The following secondary antibodies were used: goat anti-rabbit IgG Alexa Fluor 488 (1:500, Invitrogen A-11008), goat anti-rabbit IgG Alexa Fluor 546 (1:500, Invitrogen A-11010), goat anti-mouse IgG Alexa Fluor 488 (1:500, Invitrogen A-11001), and goat anti-mouse IgG Alexa Fluor 555 (1:500, Invitrogen A-21422).

### 2.3. Light Microscopy and Immunohistology

Oviduct sections were stained with antibodies against DNALI1 (Boster Biological Technologies, Pleasanton, CA, USA, A07656-1; 1:200). Testis tissues were stained with hematoxylin and eosin (H&E) dyes, to assess the absence of elongated spermatids and spermatozoa.

Testes from adult mice were fixed in 4% PFA (Electron Microscopy Sciences, 15710) in PBS overnight at 4 °C, the next day washed in 70% ethanol and embedded in paraffin. Subsequently, the tissue was sectioned (6 µm). The sections were placed in a drop of 10% ethanol on slides (Klinipath), stretched on a heating plate of 40 °C until the liquid was evaporated and dried overnight at 37 °C. The slides were stored at 4 °C for further usage. For histology, slides with sections of paraffin-embedded testes were incubated at 60 °C for 1 h. Next, the slides were deparaffinized and finally washed in 100% ethanol. Slides were then oxidized with periodic acid solution (Sigma-Aldrich, St. Louis, MI, USA, 395132) for 5 min and rinsed with Milli-Q water. Subsequently, the slides were incubated with Schiff reagent (Sigma-Aldrich, St. Louis, MI, USA, 352016) for 15 min and washed in lukewarm, streaming tap water for 5 min. Counterstaining was performed with hematoxylin (Klinipath, Olen, Belgium, 4085.9005) for 4 min, followed by washing in streaming tap water for 5 min. The slides were embedded in mounting medium (DAKO, C0563) (preheated to 45 °C). Sections with PAS staining were imaged using a Hamamatsu Nanozoomer 2.0 HT system and exported using the NDP.view software (Hamamatsu, Japan, version 2.9.25).

### 2.4. Terminal Deoxynucleotidyl Transferase dUTP Nick End Labeling (TUNEL) Analyses

Deparaffinized sections were washed 2 times for 5 min in 70% ethanol followed by 5 min in PBS. Subsequently, the slides were treated with Proteinase K (2 µg/mL in PBS) for 15 min at room temperature, followed by four 2 min washes in distilled water. Then, a 5 min incubation in 2% H_2_O_2_ in PBS was followed by a 5 min wash in distilled water. Thereafter, the slides were washed for 5 min in TdT-buffer (Promega, M1871) and incubated for 2 h in TdT-buffer containing 0.01 mM Biotin-16-dUTP (Roche, Basel, Switzerland, 11093070910) and 0.3 U/µL TdT-enzyme (Promega, M1871) at room temperature in a humid chamber. The enzymatic reaction was stopped by three 5 min washes with TB-buffer (300 mM NaCl, 30 mM tri-Natriumcitrate-Dihydrate), followed by 5 min in Milli-Q water and 5 min in PBS. Next, the slides were incubated for 10 min in 2% BSA at room temperature, followed by 5 min washes in Milli-Q water and PBS. Subsequently, the slides were incubated with ABC-HRP conjugate (Vectastain, PK-6100) for 30 min at room temperature followed again by 5 min washes in Milli-Q water and PBS. The slides were stained with DAB (DAKO, K3468) for 5 min in the dark and the staining was stopped by washing for 5 min in Milli-Q water. Counterstaining was performed with hematoxylin. The slides were embedded in mounting medium (DAKO, C0563) (preheated to 45 °C) and covered with a coverslip. Sections were imaged using a Hamamatsu Nanozoomer 2.0 HT system and exported using the NDP.view software (Hamamatsu, Japan, version 2.9.25). For quantification of TUNEL analysis, 100 round tubule cross-sections per animal were scored for TUNEL-positive cells. Per genotype, two different animals were analyzed.

### 2.5. Fluorescent Immunohistology of Testis Sections

Slides were deparaffinized and incubated in Proteinase K (1 µg/mL in PBS) for 15 min at room temperature, followed by four 2 min washes in distilled water. Epitope retrieval was performed via incubation of slides in 1× sodium citrate buffer (pH 6) for 20 min at the microwave (900 W). Slides were allowed to cool down for 1 h and then washed in PBS (3 × 5 min). Then, the slides were incubated in 10% normal goat serum (Sigma) and 5% BSA in PBS for 30 min in a humid chamber at room temperature. Incubation with the primary antibodies (diluted in 5% BSA in PBS) was performed overnight at 4 °C in a humid chamber. The next day, the humid chamber including slides was kept for 1 h at room temperature. The slides were then washed 3 times for 5 min with PBS and incubated for 1.5 h with the secondary antibody (diluted in 1% BSA in PBS) at room temperature in a humid chamber. Subsequently, the slides were washed 3 times for 5 min in PBS and embedded in Prolong Gold with DAPI (Invitrogen, Waltham, MA, USA).

### 2.6. Fluorescent Immunocytology of Meiotic Spreads

Nuclei of testicular cells were spread as previously described [29]. Immunocytochemistry on the nuclear surface spread spermatocytes was performed as previously described [30]. In short, slides were washed, blocked with BSA and milk powder, and incubated with primary antibody overnight at room temperature. The second day the slides were washed and blocked again, followed by incubation of 2 h with the secondary antibody. To finish, the slides were washed and embedded in Prolong Gold with DAPI (Invitrogen, Waltham, MA, USA). Quantification of meiotic prophase stages was performed by indicating the meiotic stage of approximately 200 nuclear surface spread spermatocytes immunostained for SYCP3. Quantification of pachytene stages was performed by indicating the pachytene stage (early, mid, or late) of approximately 100 pachytene spermatocytes immunostained for SYCP3. For MLH1 quantification, approximately 20 pachytene nuclei and 20 early diplotene nuclei images per mouse were taken using a Zeiss Axio Imager M2 including digital microscopy camera AxioCam 503. Number of MLH1 foci was counted manually. For MLH1 presence quantification, approximately 100 pachytene nuclei and 100 diplotene nuclei were scored on their presence of MLH1 foci (at least 5 foci or more counted as present). Images presented in the figures were taken using a Zeiss Confocal Laser Scanning Microscope 700× with a 40× objective (sections) or 63× objective (spreads) immersed oil. Plots were made using GraphPad Prism software (version 9). All quantifications were pooled results of two animals per genotype.

### 2.7. Transcriptome Re-Analysis

Affymetrix GeneChip HT MG-430 PM Array Plates had been previously employed to document the global transcriptome profile of CLPP-null versus WT testis (n = 3) at the age of 9–10 months [1]. Transcripts with specific functions during the subsequent spermatogenesis stages were now extracted and ordered manually, then deposited publicly on a preprint server.

### 2.8. Global Proteome Profiles by Mass Spec and Label-Free Quantification

WT and *Clpp*^−/−^ mice (n = 3 per age group) were sacrificed at postnatal days P17, P21, and P27, respectively, and their testes were dissected. Per animal, one testis was used for mass spectrometry analyses; the other one was halved and used for RNA and protein isolation.

#### 2.8.1. Sample Preparation for Liquid Chromatography Mass Spectrometry (LC–MS)

Testes from WT and CLPP-null mice collected at postnatal days P17, P21 and P27 were washed with ice-cold PBS before being resuspended in hot lysis buffer (2% SDS, 150 mM NaCl, 50 mM Tris–HCl, pH 8, 10 mM TCEP, 40 mM 2-chloracetamide, and protease inhibitor cocktail tablet (EDTA-free, Roche, Basel, Switzerland)) and passed 5 times through a 20 G needle attached to a 1 mL syringe. Lysates were incubated for 10 min at 95 °C, sonicated for 30 s with 1 s ON/1 s OFF pulse at 30% amplitude using Sonics Vibra Cell (Newtown, CT, USA), and incubated for another 10 min at 95 °C.

Sample lysates were prepared as described previously [31]. Briefly, lysates were methanol–chloroform precipitated and the protein pellets were resuspended in 8 M Urea/10 mM EPPS pH 8.2. Protein concentration was determined using the Pierce BCA protein assay kit (Thermo Fisher Scientific). Samples were diluted to 2 M urea using 10 mM EPPS pH 8.2, and digested overnight with 1:50 (*w*/*w*) ratio of LysC (Wako Chemicals, Neuss, Germany) at 37 °C. Samples were further diluted to 1 M urea and digested with 1:100 (*w*/*w*) ratio of sequencing-grade trypsin (Promega, Madison, WI, USA) for additional 6 h at 37 °C. Digests were acidified using trifluoroacetic acid to obtain a pH < 3 and purified using tC18 SepPak columns (50 mg, Waters, Milford, MA, USA). Peptides were dried and resuspended in 0.2 M EPPS pH 8.2, and 10% acetonitrile (ACN). Peptide concentration was determined using Micro BCA protein assay kit (Thermo Fisher Scientific) and 55 μg of peptide per sample was labeled with 1:2.5 (*w*/*w*) ratio of TMT 10plex labelling reagent (Thermo Fisher Scientific). A bridge channel was prepared by pooling equal amounts from all 18 samples which were TMT-labeled together and split into three sets of equimolar samples for each plex. The samples were organized across three TMT plexes such that one plex contained all three replicates for WT versus null samples for one of the three postnatal days as well as a bridge channel (used only as an additional internal control in this study). The ratios between all channels were further normalized following a single injection measurement of each plex by LC–MS/MS which was also used to control and confirm the labeling efficiency (>99% labeling of all peptide sequences for all plexes). All samples were pooled in equimolar ratios within each plex and acidified before desalting and removal of excess TMT using tC18 SepPak columns (50 mg; Waters). Peptides were dried before fractionation.

#### 2.8.2. High pH Reverse Phase Fractionation

The Dionex Ultimate 3000 analytical HPLC (Thermo Fisher Scientific) was used to perform high pH reverse-phase fractionation. For each plex, 385 μg of pooled and purified TMT-labeled samples were resuspended in 10 mM ammonium bicarbonate (ABC), 5% ACN, and separated on a 250 mm long C18 column (X-Bridge, 4.6 mm ID, 3.5-μm particle size; Waters) using a multistep gradient from 100% Solvent A (5% ACN, 10 mM ABC in water) to 60% Solvent B (90% ACN, 10 mM ABC in water) over 70 min. Eluting peptides were collected every 45 s. The resulting 96 fractions were cross-concatenated into 24 fractions and subsequently dried before LC–MS analysis.

#### 2.8.3. LC–MS

A total of 5 μg of dried peptides from each fraction were resuspended in 2% (*v*/*v*) ACN/1% (*v*/*v*) formic acid (FA) solution and 1 μg was shot. Data acquisition was performed using centroid mode on an Orbitrap Fusion Lumos mass spectrometer hyphenated to an easy-nLC 1200 nano HPLC system with a nanoFlex ion source (Thermo Fisher Scientific). A spray voltage of 2.6 kV was applied with the transfer tube heated to 300 °C and a funnel RF of 30%. Internal mass calibration was enabled (lock mass 445.12003 *m*/*z*). Peptides were separated on a self-made, 30 cm long, 75 μm ID fused-silica column, packed in-house with 1.9 μm C18 particles (ReproSil-Pur, Dr. Maisch, Ammerbuch, Germany) and heated to 50 °C using an integrated column oven (Sonation, Biberach, Germany). HPLC solvents consisted of 0.1% FA in water (Buffer A) and 0.1% FA, 80% ACN in water (Buffer B).

Individual peptide fractions were eluted by a nonlinear gradient from 5 to 60% B over 155 min followed by an increase to 95% B in 1 min and held for another 9 min. Full scan MS spectra (350–1400 *m*/*z*) were acquired using the Orbitrap with a resolution of 120,000 at *m*/*z* 200, maximum injection time of 100 ms, and automatic gain control (AGC) target value of 400,000 at *m*/*z* 200. The precursors with a charge state between 2 and 5 per full scan were selected and dependent scans were set to 10. To limit repeated sequencing of already acquired precursors, a dynamic exclusion of 45 s and 7 ppm was set and advanced peak determination was deactivated. MS2 precursors were selected with a quadrupole isolation window of 0.7 Th and fragmented by collision-induced dissociation with a normalized collision energy (NCE) of 35% and 10 ms activation time. MS2 analysis was performed in the iron trap with a rapid scan rate using a maximum injection time of 85 ms and an AGC target value of 20,000. Following acquisition of each MS2 spectrum, a synchronous-precursor-selection MS3 scan was collected on the top 10 most intense ions in the MS2 spectrum. MS3 precursors were selected with a quadrupole isolation window of 0.7 Th with multi-notch isolation. Precursors were fragmented by high energy collision-induced dissociation (HCD) with an NCE of 65% and analyzed using the Orbitrap with a resolution of 50,000 at *m*/*z* 200 with a scan range of 110–500 *m*/*z*, a maximum injection time of 86 ms, and an AGC target value of 100,000.

#### 2.8.4. Data Analysis

Raw files were analyzed using Proteome Discoverer (PD) 2.4 software (Thermo Fisher Scientific) individually for each plex. Spectra were selected using default settings and database searches were performed using the Sequest HT node in PD against trypsin digested *Mus musculus* reference isoform FASTA (UniProtKB/Swiss-Prot and UniProtKB/TrEMBL; 62,309 sequences; version 10 December 2018) and MaxQuant contaminants FASTA. Static modifications were set as TMT6 (+229.163 Da) at the N-terminus and carbamidomethyl (+57.021 Da) at cysteine residues. Methionine oxidation (+15.995 Da) was set as a dynamic modification. Search was performed using Sequest HT taking the above-mentioned modifications into account and additionally with Acetyl (+42.011 Da) modification of the N-Terminus set as dynamic modification. Precursor mass tolerance was set to 7 ppm and fragment mass tolerance was set to 0.5 Da. Default Percolator settings in PD were used to filter peptide-spectrum matches (PSMs). Reporter ion quantifications were achieved using default settings in the consensus workflow. Protein files were exported to Microsoft Excel and the unnormalized abundance values were used for further processing. Sample loading and trimmed mean of M value normalizations were performed consecutively to correct small sample loading and labeling reaction efficiency differences [32]. This was achieved by multiplying the global scaling factors to each grand total reporter ion intensity of each channel to the average total intensity across the channels using a custom script. The mean log_2_ fold changes were calculated for all quantified proteins in mutant samples with respect to their corresponding WT controls (n = 3 independent biological replicates each). Statistical significance was assessed using a two-sided, unpaired *t*-test assuming equal variance using Microsoft Excel 2016. All contaminants were removed before further analysis.

### 2.9. Quantitative PCR of mtDNA in Testis Tissues

Quantitative PCR of mitochondrial versus nuclear genes was conducted in fractionated testes of CLPP-null (*Clpp*^-/-^) and respective WT animals (n = 3, age 6–12 months). Fractionation was performed as described [33]. A total of 200 µL of each fraction (cytosolic, mitochondrial, and nuclear) were treated with proteinase K (250 µg/mL) in a buffer containing 1 M Tris/HCl pH 8.0, 5 M NaCl, 0.5 M EDTA pH 8.0, and 10% SDS) and left over night at 55 °C shaking at 500 rpm. A total of 250 µL saturated NaCl was added and the sample was shaken vigorously for 90 s and placed on ice for 10 min. After 10 min centrifugation at 5000 g, 500 µL of the upper aqueous phase was transferred to a fresh tube and mixed with 1 mL of 100 % ethanol. After 10 min of centrifugation at 16,000 g, the supernatant was removed, the pellet was washed with 70% ethanol, and the sample was resuspended in 100 µL (cytosolic and mitochondrial fraction) or 200 µL (nuclear fraction). The DNA amount was quantified using a TECAN reader (Tecan, Männedorf, Switzerland). All reactions were run in quadruplicates using a StepOnePlus real-time PCR equipment (Applied Biosystems) and SYBR Green technology using 8 ng DNA per reaction. For the amplification of the mitochondrial *Cox3* and the nuclear *Ndufv1* fragment, we used the following primers: Cox3-fwd 5′–TTTGCAGGATTCTTCTGAGC and Cox3-rev 5′–TGAGCTCATGTAATTGAAACACC, Ndufv1-fwd 5′-CTTCCCCACTGGCCTCAAG, and Ndufv1-rev-5′-CCAAAACCCAGTGATCCAGC. The relative amount of mtDNA to nuclear genome was calculated using the 2^−ΔΔCt^ method [34].

### 2.10. Reverse Transcriptase Quantitative PCR

Total RNA from tissues was extracted using TRI reagent according to the manufacturer’s protocol (Sigma, Burlington, MA, USA). DNase (Amplification Grade, Invitrogen) was applied, and SuperScriptIII (Invitrogen) was used for reverse transcription following manufacturer’s instructions. The qPCRs were performed using TaqMan gene expression assays (Applied Biosystems, Waltham, MA, USA) in cDNA from 20 ng total RNA in 20 µL reactions in a StepOneplus Real-Time PCR system (Applied Biosystems). The following assays were applied: *Adam3*: Mm00456453_m1, *Alkbh7*: Mm01232764_m1, *Aurka*: Mm01248179_g1, *Aurkc*: Mm03039428_g1, *Clpp*: Mm00489940_m1, *Dazl*: Mm012735564_m1, *Dmc1*: Mm00494490_m1, *Dnajb3*: Mm00492548_m1, *Dnajb13*: Mm00463396_m1, *Dnali1*: Mm00613749_m1, *Eral1*: Mm01742631_s1, *Hars2*: Mm00475675_m1, *Ldhc*: Mm00466648_m1, *Peo1*: Mm00467928_m1, *Poldip2*: Mm00458936_m1, *Prm3*: Mm00443095_s1, *Rec8*: Mm00490939_m1, *Stra8*: Mm00486473_m1, *Sycp3*: Mm00488519_m1, *Tbp*: Mm00446973_m1, and *Tex12*: Mm01174533_g1.

### 2.11. Subcellular Fractionation of Testis Tissues

WT and CLPP-null (n = 3 versus 3) mice were sacrificed at P21 and their testes were dissected. To distinguish different subcellular compartments, cytosolic, mitochondrial, and nuclear fractions were prepared by differential detergent extraction, as previously published [33]. The protein content was determined using BCA assay (Life Technologies, Carlsbad, CA, USA, #23227).

### 2.12. Quantitative Immunoblots

Whole protein extracts from tissues were obtained using RIPA buffer, supplemented with protease inhibitor (cOmplete, Mini Protease Inhibitor Cocktail, Merck Millipore, Darmstadt, Germany, #4693159001) as described before [15]. Protein lysate aliquots of 12.5 µg from either whole protein or subcellular fractions were loaded on SDS gels and quantitative immunoblots were performed as described earlier [1]. Membranes were incubated with the following primary antibodies: acetyl-α-Tubulin (Cell Signaling Technology, Danvers, MA, USA, #9272; 1:1000), acetyl-Histone H3K9 (Cell Signaling, #9649; 1:1000), acetyl-SMC3 (Sigma, MABE1073; 1:1000), cGAS (Cell Signaling, #31659, 1:1000), CLPP (Proteintech, Manchester, UK, 15698-1-AP; 1:1000), CLPX (Invitrogen, PA5-79052; 1:1000), DNAJA3 (Santa Cruz, Dallas, TX, USA, sc-46588; 1:1000), ERAL1 (Proteintech, 11478-1-AP; 1:1000), GRSF1 (Sigma, HPA036985; 1:1000), HARS2 (Proteintech, 11301-1-AP; 1:1000), Histone H3 (Abcam, Cambridge, UK, ab1791; 1:1000), PEO1 (Abcam, ab187517; 1:1000), RMND1 (Abcam, ab223119; 1:1000), TFAM (Merck Millipore, ABE483, 1:1000), and Tubulin (Cell Signaling, #2144; 1:1000 or Sigma, #9026, 1:1000). Loading control was achieved with GAPDH (Calbiochem, Merck Millipore, CB1001; 1:1000) for cytosolic fractions, HSP60 (SantaCruz, sc-13115; 1:500) for mitochondrial fractions, or LAMIN A/C (Abcam, Cambridge, UK, ab169532; 1:1000) for nuclear fractions, and TBP (Abcam, ab63766; 1:500) for whole protein extracts.

### 2.13. cGAMP ELISA

Approximately, 50 mg of testis (n = 9, 5- to 12-month-old male mice) tissue was crushed and lysed in 1% NP-40 lysis buffer, then spun down at 15,000 rpm for 10 min at 4 °C. The supernatant was collected as protein lysate and quantified with micro-BCA assay (#23235, Proteintech). Undiluted protein samples were used to measure cGAMP concentrations using a 2′3′-cGAMP ELISA Kit (Cayman Chemicals, Ann Arbor, MI, USA #501700) in accordance with the manufacturer’s protocol.

### 2.14. Statistical Analyses

Statistical analyses and heatmap generation were performed using Graphpad Prism software versions 8 and 9. Bar graphs show variances as the standard error of the mean (SEM) and *p*-values from Welch’s *t*-test (T 0.05 < *p* < 0.1; * *p* < 0.05; ** *p* < 0.01; *** *p* < 0.001; **** *p* < 0.0001).

## 3. Results

### 3.1. Analyses of Male Meiotic Propase Progression in CLPP-Null Mice

**Figure 1:**
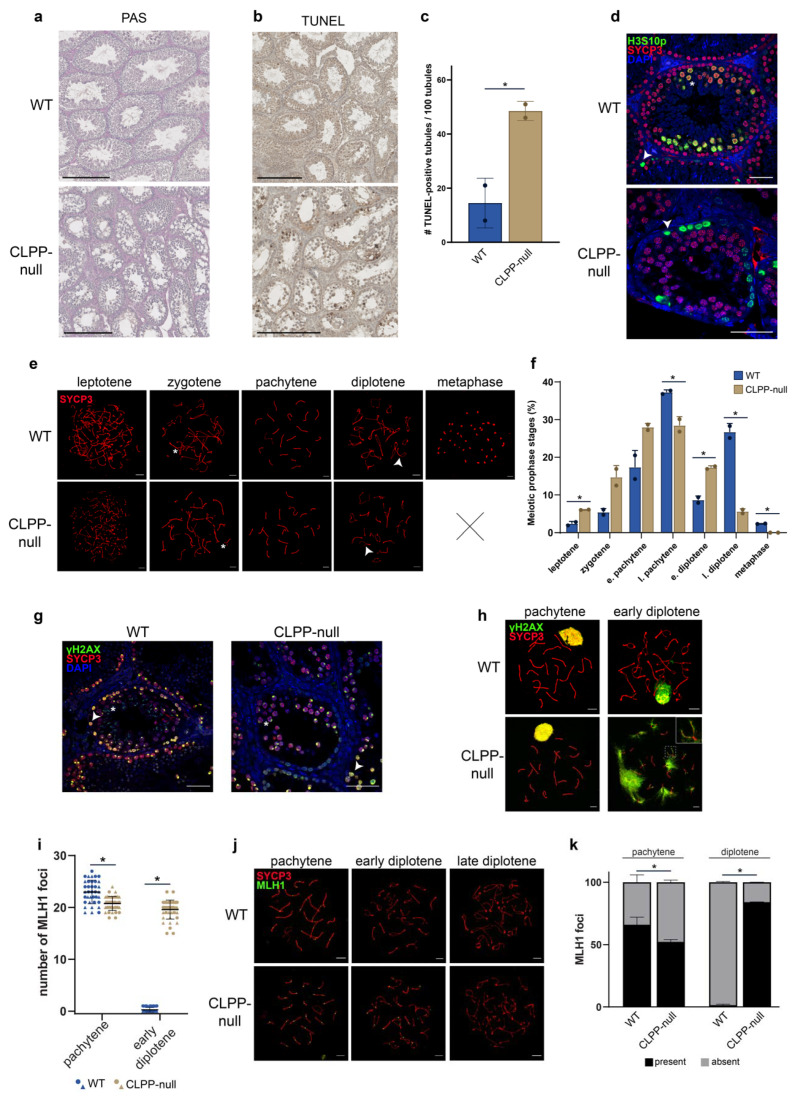
(**a**) PAS staining on sections of 8-week-old testes of WT and CLPP-null mice. (**b**) TUNEL staining on sections of 7-week-old testes of WT and CLPP-null mice. (**c**) Quantification of the number of TUNEL positive tubules per 100 tubules for WT and CLPP-null testes (200 tubules of two different animals per genotype; * indicates *p* < 0.05 (unpaired *t*-test, n = 2 animals of each genotype)). (**d**) Representative image of immunohistochemistry of H3S10p (green), SYCP3 (red), and DAPI (blue) on sections of WT and CLPP-null testes. Asterisk indicates a SYCP3^+^/H3S10p^+^ cell and the arrowhead indicates a SYCP3^−^/H3S10p^+^ cell. (**e**) Representative images of spread spermatocyte nuclei immunostained for SYCP3 (red), showing the different meiotic prophase stages present in WT and CLPP-null testes. Asterisk indicates chromosome which is partly synapsed, and arrowhead indicates region that is desynapsing. (**f**) Quantification of meiotic prophase stages on meiotic spread nuclei of WT and CLPP-null testes e.; early, l.; late, (427 nuclei and 429 nuclei from two different animals, respectively; * indicates *p* < 0.05 (unpaired *t*-test, n = 2 animals of each genotype)). (**g**) Representative image of immunohistochemistry for γH2AX (green), SYCP3 (red), and DAPI (blue) on sections of WT and CLPP-null testes. Asterisk indicates a late prophase spermatocyte and the arrowhead indicates an early prophase spermatocyte. (**h**) Representative images of pachytene and early diplotene spread spermatocyte nuclei of WT and CLPP-null testes immunostained for γH2AX (green) and SYCP3 (red). Region within the dotted square is enlarged in the inset. γH2AX specifically accumulates along desynapsed regions, as shown more clearly in the enlargement. (**i**) Quantification of MLH1 foci number on meiotic spread preparates of WT and CLPP-null testes in pachytene (37 and 39 nuclei, respectively, from two different animals per genotype, * indicates *p* < 0.05 (unpaired *t*-test)) and early diplotene stage (39 and 41 nuclei, respectively, from two different animals per genotype, * indicates *p* < 0.05 (unpaired *t*-test)). Symbol shapes represents individual animals. (**j**) Representative images of pachytene, early diplotene, and late diplotene spread spermatocyte nuclei of WT and CLPP-null testes immunostained for MLH1 (green) and SYCP3 (red). (**k**) Quantification of MLH1 foci presence on meiotic spread preparates of WT and CLPP-null testes in pachytene (203 and 201 nuclei, respectively, from two different animals per genotype; * indicates *p* < 0.05 (Chi-square test)) and diplotene stage (200 and 201 nuclei, respectively, from two different animals per genotype; * indicates *p* < 0.05 (Chi-square test)). Blue color represents WT and taupe color represents CLPP-null (**c**,**f**,**i**). Scale bar represents 250 µm (**a**,**b**), 50 µm (**d**,**g**), and 5 µm (**e**,**h**,**j**).

Previously, it was described that CLPP-null male mice displayed a complete absence of mature spermatids and spermatozoa, explaining the early complete infertility of this mutant mouse that models PRLTS3 [1]. However, the exact defects that may occur during meiosis and cause the lack of further germ cell development were not established. Therefore, we now analyzed spermatogenesis and in particular meiotic prophase in more detail. Periodic acid-Schiff (PAS) staining on testis sections to mark acrosomes confirmed the previous data showing a lack of spermatids and spermatozoa in CLPP-null male mice (Figure 1a). We next performed terminal deoxynucleotidyl transferase dUTP nick end labeling (TUNEL) analyses and observed more than a doubling of the frequency of tubules containing apoptotic nuclei in the absence of CLPP (Figure 1b,c). Since no post-meiotic cells were observed, we inferred that the apoptotic nuclei must represent spermatocytes (primary or secondary). To precisely identify the stage(s) of spermatogenesis at which the cells died, we studied the progression of meiotic prophase in more detail, using an antibody against the synaptonemal complex protein SYCP3, which marks the axial and lateral elements of the SC, the protein structure that connects the chromosomal axes of homologous chromosomes once they pair. In addition, we stained for Histone 3 Serine 10 phosphorylation (H3S10p), a histone modification that marks chromosomes when cells enter either mitotic or meiotic metaphase [35]. In WT testis sections, we observed different substages of meiotic prophase, and also nuclei that were positive both for SYCP3 (which remains on the region around the centromeres in meiotic metaphase [36]) and H3S10p, marking the cells in meiotic metaphases I and II, in tubules that can be categorized as Stage XII according to Oakberg [37]. In the absence of CLPP, we also observed different meiotic prophase substages, but all H3S10p-positive nuclei lacked SYCP3 signal, indicating that there are no meiotic metaphases in the CLPP-null male mice (Figure 1d). We further assessed to what extent meiotic prophase progressed normally in terms of chromosome pairing and synapsis using spread meiotic nuclei, which allows for an easier assessment of the phases of chromosome pairing and substaging of the nuclei into leptotene (fragments of axial elements are formed), zygotene (initiation of synapsis), pachytene (synapsis is completed), and diplotene (desynapsis and gradual removal of the SC). In CLPP-null spermatocytes, chromosome synapsis and desynapsis appeared to occur normally, and again, no meiotic metaphases were observed (Figure 1e). Additionally, another component of the axial elements of the SC, HORMAD1, displayed a normal staining pattern in CLPP-null spermatocyte nuclei (Figure S1a). The quantification of the frequencies of nuclei at the different meiotic prophase stages showed increased frequencies of leptotene and early diplotene spermatocytes, while late pachytene and diplotene spermatocyte numbers were reduced, and metaphases were not observed (Figure 1f). Altogether, this indicates that spermatogenesis arrests at the end of meiotic prophase-I, during late diplotene, but before entry into metaphase in the absence of CLPP.

Next, we assessed the formation and repair of meiotic DSBs, detecting the ssDNA binding protein complex RPA, which forms foci at sites of meiotic DSB repair [38]. We observed no differences in the pattern of RPA foci formation and disappearance between wild-type and CLPP-null nuclei, indicating that the progress of homologous recombination repair occurred in a normal fashion, so that all foci are removed by the end of pachytene (Figure S1b). We also assessed the formation of the XY body, as another marker for progression of chromosome pairing and DSB repair [39]. The XY body represents the chromatin domain of the X and Y chromosomes. This chromosome pair displays stable synapsis only in the short region of homology, the pseudoautosomal region. The large unsynapsed chromosome arms, with unrepaired meiotic DSBs, are thought to trigger the formation of the transcriptionally silent XY body, which is marked by an intense accumulation of the phosphorylated form of H2AX (γH2AX) [39]. The γH2AX immunostaining signal first accumulates in the chromatin surrounding all meiotic DSBs in leptotene and zygotene, but is observed only on the XY body in mid-pachytene. We observed no obvious differences in this pattern between wild-type and CLPP-null testis sections (Figure 1g), and also not in meiotic spreads of spermatocytes up to pachytene (Figure 1h and Figure S2). However, to our surprise, γH2AX re-accumulated in CLPP-null diplotenes, emanating as flares from the SC (5.86 ± 3.9 flares per nucleus), in particular along the asynapsed regions (92% of 252 flares on desynapsed versus synapsed regions from 43 nuclei of two animals) (*p* < 0.005, Z-test). The number of flares was very variable, and some CLPP-null diplotene nuclei also lacked them (Figure S2). Still, the difference with WT diplotenes was clearcut (0.38 ± 0.74 flares per nucleus) (*p* < 0.05, unpaired *t*-test) (Figure 1h and Figure S2). To further understand the progression of meiotic DSB repair, we next tested whether meiotic DSB repair progressed into the formation of crossovers, which can be detected using an antibody that recognizes the mismatch repair protein MLH1 in mid-to-late pachytene spermatocytes [23]. Surprisingly, immunocytochemistry on meiotic spreads revealed that MLH1 foci were present in both pachytene and diplotene of the mutant, while this was not the case in WT, where the vast majority of MLH1-positive spermatocytes where in pachytene (Figure 1i–k and Figure S3). The average number of MLH1 foci per nucleus was also slightly decreased (*p* = 0.02, unpaired *t*-test) in CLPP-null spermatocytes. The presence of MLH1 foci in diplotene could be caused by: (1) persistent MLH1 foci in diplotene (problems with removal of MLH1), (2) precocious start of desynapsis while MLH1 has not yet been removed, or (3) delayed formation of MLH1 foci. If diplotene is initiated normally, but the foci persist, it would be expected that the relative percentages of pachytene nuclei with and without MLH1 foci would be similar for control and CLPP-null mutants, but instead, the fraction of pachytene nuclei with MLH1 foci was reduced (Figure 1k). This would be consistent with delayed MLH1 focus formation or precocious entry into diplotene. If foci formation is delayed, the relative distribution of early and late pachytenes would be expected to be similar between the two genotypes, but instead, fewer late pachytenes, and more early diplotenes were observed in the CLPP-null mice (Figure 1f). These findings are consistent with premature desynapsis occurring in the CLPP-null spermatocytes, in particular because synapsis and DSB repair proceed normally in the CLPP-null spermatocytes, with complete removal of RPA and γH2AX from the autosomes. In combination with the reduction in late diplotene frequency and absence metaphase-I nuclei and later stages, we propose that CLPP-null spermatocytes desynapse too early compared to their WT equivalents, and eventually undergo cell death during late diplotene.

### 3.2. Global Proteome Data Analysis in Juveniles Recapitulates Lack of Progression to Metaphase-I in CLPP-Null Mice

**Figure 2:**
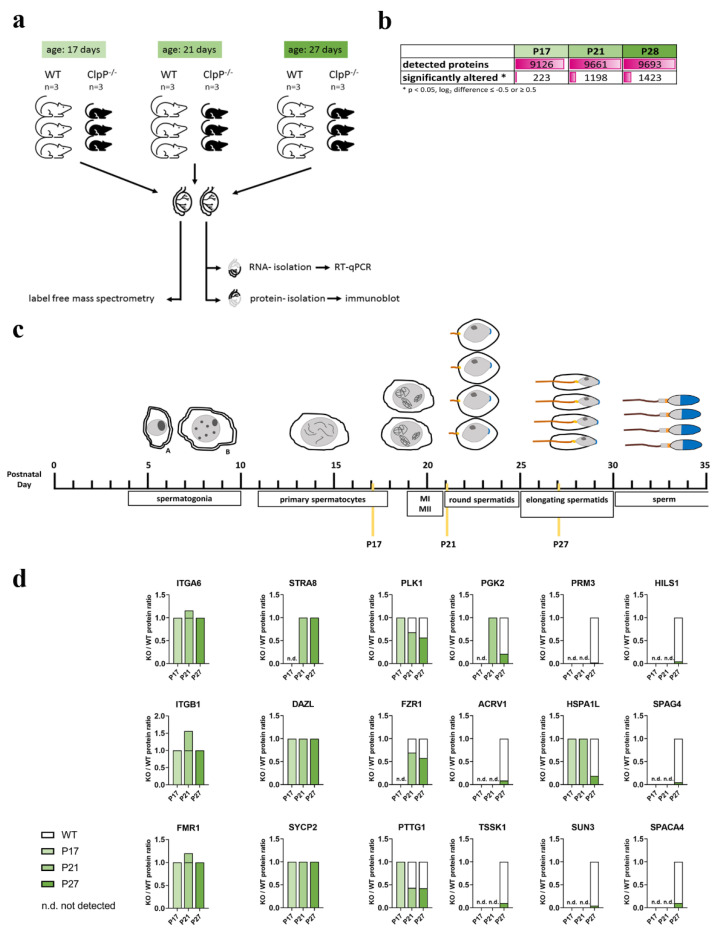
(**a**) Schematic design of breeding, dissection, and extraction of protein and RNA for molecular profiling at three age points. (**b**) Number of successful protein quantifications and of significant dysregulations with relevant effect size (log2 difference ≤−0.5 or ≥0.5) is shown for each age point (shades of green reflect age progression). (**c**) Scheme according to [41,42], illustrating what differentiation stages were studied within the almost synchronous spermatogenesis during the first 27 postnatal days of mice. (**d**) Mass spectrometry label-free quantification of specific proteins known as markers of successive maturation stages during spermatogenesis. Boxes are normalized to WT, the *Y*-axis value 1 representing 100%, and a partially green box suggests fewer numbers of cells reaching this stage. A moderate reduction was first observed at P21 for late spermatocyte markers PLK1, FZR1, and PTTG1. A massive reduction was observed at P21 for round spermatid markers PGK2, ACRV1, and TSSK1, while a total loss was observed at P27 for elongating spermatid markers PRM3, and SUN3, as well as for sperm markers HILS1, SPAG4, and SPACA4. n.d. = not detected.

From the microscopy analyses of spermatogenic progression described above, it is to be expected that a relative loss of factors with a role in late meiotic prophase and/or thereafter would be observed in the transcriptomic and proteomic data of mutant mice, due to the absence of later maturation stages from total testis homogenate. Conversely, genes that are highly expressed in spermatogonia or early spermatocytes would be expected to be overrepresented in adult CLPP-deficient testes, due to the relative enrichment of these cell types. This notion is confirmed by the previously reported downregulation of many spermatid-specific transcripts [1], and the upregulation of early meiotic transcripts such as *Dmc1, Spo11*, *Hormad1*, *Hormad2*, *Sycp2*, *Sycp3*, and *Meiob* [40]. Now, we set up a hypothesis-free systematic analysis of proteome profiles, focusing on juvenile testis with the separate investigation of three ages: postnatal day P17, when the most advanced cells should be almost entering the M-phase of the first meiotic division; P21, when the two meiotic divisions have just been completed in the most advanced cells; and P27, when the first elongating spermatids are formed. One testis per mouse was used for protein extraction and mass-spectrometry (MS) with label-free quantification of individual peptides, while the other organ was destined for RNA extraction to perform RT-qPCR, and for protein extractions to generate immunoblots (see Figure 2a). Overall, almost 10,000 proteins could be quantified in each MS analysis by the tandem mass tag (TMT) approach, and significant dysregulations were encountered for 223 factors at P17, 1198 factors at P21, and 1423 factors at P27 (Figure 2b, Supplementary Table S1).

Before the attempt to define selective pathway impairments, it is crucial to control the percentage of cells reaching individual stages of spermatogenesis and to interpret any molecular dysregulation relative to cell numbers. Established protein markers for each maturation state were compared for CLPP-null versus WT testis at each age point (Figure 2c,d). The spermatogonia markers ITGA6 (CD49F), ITGB1 (CD29), and FMR1 [43] showed slight increases. Early spermatocyte prophase-I markers STRA8, DAZL, and SYCP2 [43,44] had normal protein amounts at all ages. In contrast, metaphase/anaphase markers PLK1, FZR1, and PTTG1 [45,46,47,48] at P21 and P27 varied between 40% and 70%, consistent with our observation that spermatocytes do not proceed beyond diplotene and fail to enter M-phase. Accordingly, late spermatocyte and round spermatid markers PGK2, ACRV1, and TSSK1 [49,50] at P27 showed abundance diminished to 10% or 20%, and the loss of post-meiotic cells was even more strikingly visualized by the dramatic decrease (5% of wild-type) in measured levels of elongating spermatid (PRM3, SUN3, and even the relatively unspecific molecular chaperone HSPA1L) and mature sperm markers (HILS1, SPAG4, and SPACA4) at P27.

Overall, the proteome profiles support the cytological observations that spermatocyte development does not proceed beyond diplotene.

### 3.3. Identification of Main Affected Pathways in Juvenile Clpp^−/−^ Testes through Proteome and RT-qPCR Analyses

**Figure 3:**
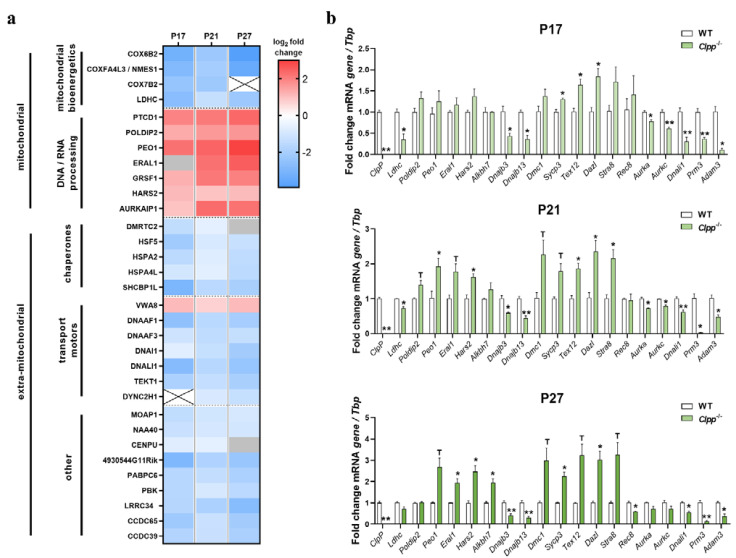
**Consistency and progression of proteome and transcript dysregulations during spermatogenesis.** (**a**) Pathways with significant enrichment of dysregulations in the proteome profile of three differentiation stages are selected, with prominent protein components shown as heatmaps with a color gradient illustrating the fold-change (red for upregulation, blue for downregulation). Crossed fields indicate non-detection of that protein, grey fields indicate non-significant changes at that stage. (**b**) Analysis of transcript levels at the three differentiation stages for key molecules in each pathway usually shows concordant dysregulations to underlie the changed protein abundance, with decreased expression mostly evident already at P17, well before the assembly into differentiated structures takes place. * *p* < 0.05, ** *p* < 0.01, T represents 0.05 < *p* < 0.10.

To define the affected pathways that may be most directly related to the absence of CLPP, we focused on significant changes that were already apparent at P17 and P21. As known correlates of the mitochondrial impact on asthenozoospermia, early and stable decreases in abundances were observed for bioenergetics proteins COX6B2, COX7B2, LDHC, and LDHAL6B (illustrated by heatmaps in Figure 3a, Table S1). The decrease in stress response factor COXFA4L3 (also known as NMES1/MOCCI/C15ORF48) reflects a mitochondrial adaptation within antiviral immunity signaling [51,52]. Finally, we observed a marked and consistent upregulation of the mitoribosomal subunit MRPS38 (also known as AURKAIP1) (Table S1).

As reported in [17], this immune activation of CLPP-null cells responds to the release of toxic mtDNA and mtRNA into the cytoplasm, and in testis, the early and stable accumulations of mtDNA/mtRNA binding mitochondrial proteins such as PTCD1, POLDIP2, GRSF1, ALKBH7, and mitochondrial PRLTS proteins PEO1, ERAL1, and HARS2 (Figure 3a, Table S1) reflected this pathology. Overall, the vast majority of significant, and more than two-fold upregulated proteins were mitochondrial components, or directly related to mitochondrial function (35 out of 44).

Extra-mitochondrially, outstanding downregulations already at P17, and maintained thereafter, included a strong reduction in the chaperone HSPA2 (34%) and its interactor protein SHCBP1L (12%), which were both suggested to be involved in chromosomal desynapsis and spindle integrity in male germ cells [53,54,55] (Table S1). The other most extremely downregulated gene was the uncharacterized 4930544G11Rik protein (with a Rho GTPase domain) (13%, Table S1). Strong reductions were also found for the transcription factors HSF5 (to 5% at P17, 26% at P21, 18% at P27) and DMRTC2 (to 15%, 34%, and 37%, respectively) (Table S1). Interestingly, HSF5 is essential for progression through meiotic prophase in zebrafish spermatogenesis [56], and DMRTC2 (also known as DMRT7), which is a member of a transcription factor family that plays prominent roles in sex determination and sex differentiation [57], has been shown to be essential for the progression of meiotic prophase beyond pachytene [58]. Two additional testis-enriched proteins, the RNA binding protein PABPC6, and the kinase PBK levels were also reduced to below 30% of WT from P17 [59,60] (Table S1). Finally, several factors related to ciliopathies such as DNALI1 [61], DNAAF1 [62], CCDC65 [63], CCDC39 [64], and LRRC34 [65] were strongly reduced already at P17 (14–31% of wild-type level, see Table S1). All of these factors are expressed the highest in post-meiotic spermatids, but also at almost equally high levels in spermatocytes, based on single-cell RNAseq data of human testes available through the Human Protein Atlas website (https://www.proteinatlas.org/, accessed on 13 December, 2020). Next, we validated the notion that early spermatogenesis stages are overrepresented in the mutant tissue, using RT-qPCR validation experiments, and indeed observed an upregulation for the meiosis inducer *Dazl* (to 235% at P21) and the meiosis inducer *Stra8* (216% at P21) (Figure 3b), as well as several SC/cohesion/HR factors from P17 (*Sycp3*, *Tex12*). Conversely, the cohesin component *Rec8* was decreased, presumably due to the high expression of the mRNA in mouse spermatids [66]. In addition, the decreases inf *Aurka* and *Aurkc* (Figure 3b) may be due to their highest levels in very late (diplotene/diakinesis/M-phase-I) spermatocytes [67,68], which are also lost. All other upregulations and downregulations could also be attributed to the relative overrepresentation of early spermatogenesis stages or to the loss of post-meiotic cells in testis tissue, respectively, or were not consistent through development.

As one of the main findings of this study into the underlying mitochondrial pathogenesis, the proteome profile revealed CLPP loss to cause early and consistent accumulation of several PRLTS disease proteins (illustrated by volcano plots in Figure S4). In the heatmap (Figure 3a) among DNA/RNA processing factors, the early and consistent accumulation of mitochondrial factors was evident, including PEO1, ERAL1, and HARS2, whose mutations are known to trigger Perrault syndrome with ovarian germ cell failure. While CLPP mutations cause PRLTS3, mutations in PEO1, ERAL1, and HARS2 are responsible for PRLTS5, PRLTS6, and PRLTS2, respectively. PEO1 (also known as TWNK) has a DNA-helicase and a primase domain that preferentially acts at the D-loop of mtDNA [69,70], so if excess amounts of small PEO1 isoforms redistribute to the nucleus, they may somehow interfere with meiotic homologous recombination repair. ERAL1 acts as an rRNA chaperone during mitoribosomal biogenesis [71], and its impact on DNA processing is presumably indirect, but it is important to know that mitoribosomal biogenesis is connected with mtDNA replication by the parallel expression co-regulation of MRPL43 and PEO1/TWNK by a joint promoter [72,73]. HARS2 is known as the mitochondrial amino acid synthetase for histidyl-tRNA, which is co-processed with serine-tRNA in a methylation complex involving MRPP3/PRORP, as a further putative Perrault syndrome protein [11,74,75,76]. Although CLPP deficiency is thought to impair mitochondrial protein degradation and the excess abundances of PEO1, ERAL1, and HARS2 may be expected to trigger a negative feedback effect on transcription, we observed a significant increase in mRNA levels of these genes at P21 and/or P27 (Figure 3b). It is conceivable that the elevated levels of these transcripts are simply due to a relative increase in early spermatocytes in the CLPP-null testis homogenate, or that the induction of these PRLTS factors is needed to compensate for CLPP dysfunction.

### 3.4. Microscopy and Quantitative Immunoblot Validation of Prominent Pathway Alterations Leading to Block

Sperm tail formation was completely absent in previous microscopy of CLPP-null mice [1], but the reductions in LRRC34 and several dynein components already at P17 stood out, so we tested whether a general ciliogenesis problem is apparent. This may also explain the progressive hearing loss of CLPP-null mice since inner ear hair cells depend on cilia function. Therefore, the motile cilia in the oviduct of female CLPP-null mice were studied histologically and with DNAI1 immunohistochemistry, but appeared normal (Figure S5).

**Figure 4:**
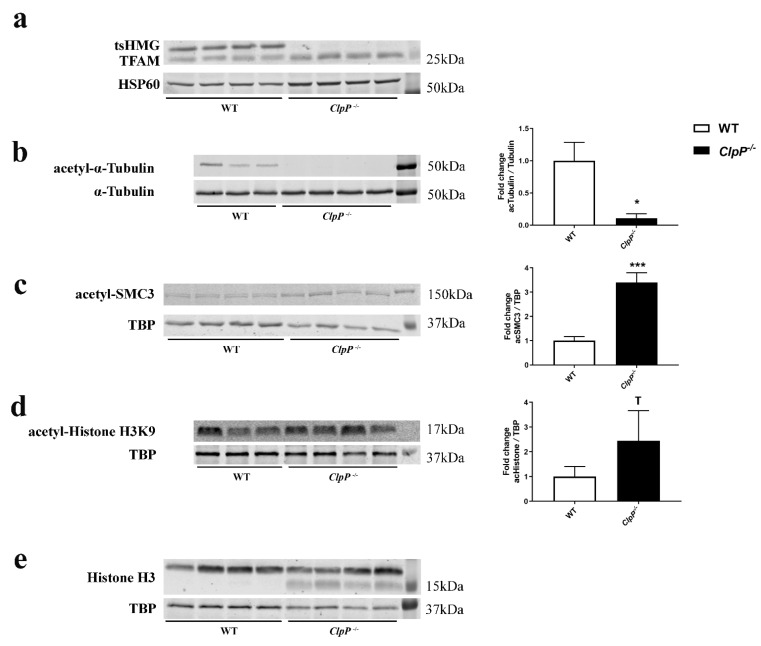
**Quantitative immunoblots of whole protein from testis tissues.** RIPA fractions from 5-month-old WT and matched CLPP-null testis were analyzed for (**a**) TFAM with its testis-specific isoform (tsHMG), normalized against mitochondrial HSP60, (**b**) acetyl-α-Tubulin, normalized against α-Tubulin, (**c**) acetyl-SMC3, (**d**) acetyl-histone H3K9, or (**e**) Histone H3. In (**a**,**e**), quantification was not performed, since zero signal would provide unrealistic effect sizes. * *p* < 0.05, *** *p* < 0.001, T represents 0.05 < *p* < 0.10.

To further validate the impaired pathways and to elucidate the involvement of mitochondria, quantitative immunoblots were performed next. The mtDNA-binding transcription factor TFAM in mice has a testis-specific isoform known as tsHMG that localizes only to the nucleus and is produced from the elongated spermatid stage onward [77]. Immunoblots confirmed the complete absence of this nuclear TFAM isoform (Figure 4a).

Given that mitochondrially generated acetyl-CoA enables microtubule assembly for spindle dynamics, cell polarization anchored at the two centrioles, and axonemal tail formation, we studied the ratio of acetylated tubulin versus total tubulin (Figure 4b). A massive decrease was observed in 5-month-old testis, probably reflecting the loss of sperm tails due to spermatid absence. This decrease was not observed yet at P17 and P21 (Figure S6a), suggesting that the microtubules of the nuclear spindle apparatus are not affected at this stage and that anaphase deficits cannot be explained by a deficit in acetyl–tubulin. Again, 12-month-old brain tissue showed no acetyl–tubulin change, indicating that sufficient acetyl–CoA is available despite CLPP-dependent mitochondrial dysfunction.

In view of the importance of acetylation at K105/K106-SMC3 to stabilize the cohesin complex and enable sister-chromatid cohesion [78], acSMC3 levels were also evaluated. The immunoblots revealed a >3-fold upregulation in 5-month-old testis (Figure 4c), providing evidence that the acetyl–CoA levels are not restricted by CLPP-dependent mitochondrial dysfunction and that the cohesin complex is sufficiently locked for meiosis-I and HR to occur correctly. The investigation of acSMC3 levels in testis at P17 and P21 were consistent with this finding, although the low sample number precluded significance (Figure S6b). These findings are compatible with the notion that elevated acSMC3 levels mirror the leptotene–pachytene spermatocyte overrepresentation in testis homogenate.

In view of the accumulation of mitochondrial ALKBH7 as a demethylase at P21 (and similar trend at P17 and P27) (see Figure 3a), we next assessed one-carbon metabolism with methylation pathology, also because this is part of the mitochondrial integrated stress response, particularly upon Twinkle mutation and mitoribosomal translation changes [79,80,81]. Waves of methylation at different histone lysine positions are a prerequisite for subsequent stages of spermatogenesis. Quantitative immunoblots detected only faint smeared bands of H3K9–methyl epitopes without an evident genotype-dependent difference, but the reciprocal acetylation of H3K9 showed a trend of increase (Figure 4d). Similarly, H3K4 methylation immunoblots detected only faint smeared bands of appropriate size in Tricine gels without obvious CLPP-dependent differences. Serendipitously during these experiments, the immunoblots of total H3 revealed the appearance of a smaller isoform in CLPP-null samples (Figure 4e), of a size known for H3 proteolytic cleavage as cellular stress response, e.g., upon virus infection or DNA damage [82,83].

It is conceivable that nuclear anomalies such as cleavage of histone H3 and the mitochondrial deficit of COXFA4L3/NMES1 may constitute events within the antiviral program upon pathological extrusion of mtDNA/mtRNA or the translation stress with colliding mitoribosomes [84] via the cGAS-STING signaling pathway. Therefore, the mtDNA gene *Cox3* was quantified versus the nuclear-encoded gene of the mitochondrial respiratory chain subunit *Ndufav1*, in three subcellular fractions enriched, respectively, for mitochondria, cytosol, and nucleus. This qPCR analysis showed a significant increase (*p* = 0.005) in ectopic mtDNA in the cytosol (Figure S7a), where it would trigger innate immunity. Consequently, the levels of cGAMP were quantified with the ELISA technique. cGAMP is an intracellular second messenger synthesized by the nucleotidyltransferase cGAS in response to cytosolic double-stranded DNA that binds STING and stimulates the production of type I interferons [85]. CLPP-null testis showed a significant increase (*p* < 0.005; almost 1.5-fold) in cGAMP levels (Figure S7b), consistent with our previous findings of potentiated type I interferon responses and cGAS-STING signaling in various tissues and cells of CLPP-null mice [1,16]. Given that cGAS is normally kept inactive by tight association with nuclear histones, but can redistribute to the cytosol when toxic DNA appears there or ribosomes collide [84,86], testis fractionation by differential detergent extraction was used to assess cGAS localization, using GAPDH to control the purity of the cytosolic fraction. Unexpectedly, the immunoblot demonstrated an exclusively nuclear presence of cGAS also in CLPP-null testis (Figure S7c), compatible with the idea that the pathological mtDNA/mtRNA extrusion exerts its toxicity in the nucleus rather than the cytosol.

However, the ablation of STING and type I interferon signaling by genetic deletion of downstream innate immune mediators STING and IFNAR failed to rescue infertility and testis involution, as shown by the lack of mature sperm in the double-mutant CLPP-null/STING-KnockOut (KO) and CLPP-null/IFNAR-KO testis (Figure S7d,e). Thus, a straightforward therapeutic avenue for CLPP-null infertility by anti-inflammatory intervention could not be substantiated. However, these results cannot completely rule out a relevance of innate immunity for fertility, since there may be a type I interferon-independent role for cGAS activation and cGAMP accumulation in CLPP-null testis. Beyond its role in innate immunity, cGAS has been implicated in several cellular processes, such as genomic instability, DNA damage responses, and cellular senescence [87,88]. Some of the anomalies in meiosis observed in CLPP-null testis could result from aberrant cGAS activation downstream of mtDNA instability. Future work is required to explore the mechanistic details behind this possibility.

### 3.5. Redistribution of Excess Mitochondrial Proteins to the Nucleus Possibly Contributes to Pathway Alterations

**Figure 5:**
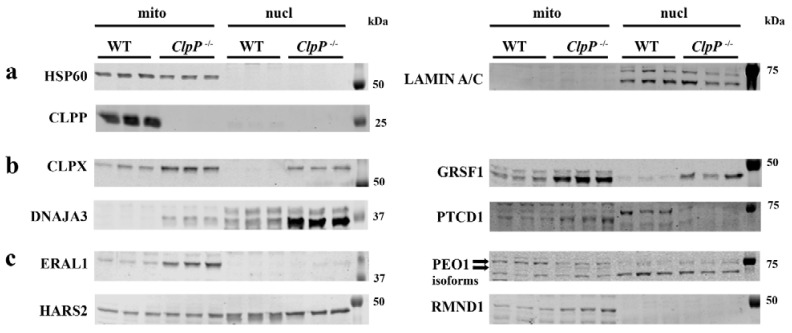
**Excess of mitochondrial proteins can trigger redistribution to nuclear fraction.** Testis P21 was fractionated with differential detergents and protein extracts were analyzed by quantitative immunoblots, normally blocked with BSA. (**a**) Fractionation purity was controlled by mitochondrial marker HSP60 and nuclear membrane markers LAMIN-A/C, sample genotype was controlled by assessment of CLPP presence. (**b**) Evident relocalization from mitochondria to nuclei was again observed for excess CLPX and its interactome component GRSF1, as well as the mature small isoform TID1S of DNAJA3. In contrast, the accumulation occurred purely in mitochondria for PTCD1. (**c**) Immunoblots confirmed CLPP to modulate the abundance of several PRLTS proteins. ERAL1 was accumulated in the mitochondrial fraction, and a faint band was apparent in mutant nuclear fractions. In contrast, PEO1 detection with a commercial rabbit polyclonal antibody directed against the C-terminal half showed a decrease in full-length 77 kDa Twinkle band with the parallel accumulation of 66 kDa Twinky band (arrows on the left side). A nuclear redistribution was not detected for any PEO1 isoform. HARS2 immunoreactivity of appropriate molecular weight was observed in the nucleus even for WT samples, with strong accumulation for CLPP-null samples. The assessment of RMND1 also suggested mitochondrial accumulation, but no presence in the nucleus.

Since the inflammation pathway data did not clarify why CLPP deficiency impacts meiosis massively in contrast to mitosis, we next focused on the accumulation of three PRLTS proteins in mitochondria of CLPP-null testis, whose mutations also disable germ cell differentiation. In CLPP-mutant fibroblasts, we previously observed that the mitochondrial release of accumulated toxic mtDNA/mtRNA is accompanied by the extrusion of their associated proteins such as STAT1 to the cytosol and DNAJA3/GRSF1/GFM1 to the nucleus [15,17]. To test whether also in testis the accumulated mitochondrial DNA/RNA processing proteins are redistributed and may contribute to nuclear pathology, tissue was extracted with differential detergents to obtain the mitochondrial, cytosolic, and nuclear fraction, at P21 when meiosis I should be completed for the first spermatogenic cohort. Fraction purity was validated with HSP60 as mitochondrial matrix marker and LAMIN-A/C as nuclear membrane markers, verifying also the complete loss of CLPP from mutant samples (*p* = 0.0014) (Figure 5a). CLPX and GRSF1 were detected at the expected sizes (predicted 69 kDa and 53 kDa for the precursor proteins, respectively), their excess abundance in CLPP-null samples was confirmed (mitochondrial CLPX 3.9-fold, *p* = 0.0192, mitochondrial GRSF1 6.6-fold, *p* = 0.0001), and their redistribution to the nuclear fraction was also apparent, although without significance in view of the low sample number and high variance (Figure 5b), as previously reported in CLPP-null MEFs [15]. Similarly, the accumulation of DNAJA3 in mitochondria led to the massive relocalization of its small isoform TID1S (49 kDa as opposed to the full-length protein with 52 kDa) [89] to the nuclear compartment (ten-fold, *p* = 0.1524) (Figure 5b). PTCD1 accumulation appeared relevant given its impact on 3′ end processing of mitochondrial tRNAs and on the repression of mitochondrial leucine tRNA, with its abundance in inverse correlation to COX activity [90,91]. This pathway is key to the pathomechanism triggered by LARS2 mutations that result in PRLTS. Immunoblot quantification documented PTCD1 abundance to increase significantly (mitochondrial PTCD1 4.0-fold, *p* = 0.0137), without nuclear relocalization (Figure 5b).

Commercial antibodies with sufficient sensitivity and specificity to detect dysregulations of endogenous ALKBH7, AURKAIP1, or VWA8 could not be identified.

ERAL1 as an rRNA chaperone is responsible for PRLTS6. In the mitochondrial fraction, immunoblots demonstrated its accumulation (9.8-fold, *p* = 0.0004), while the nuclear fraction of CLPP-null samples showed a subtle accumulation without significance given the limited statistical power (Figure 5c). PEO1 as the protein responsible for PRLTS5 failed to show the prominent accumulation detected by mass spectrometry (Figure 5c), and exhibited no nuclear localization in the testis, despite an immunocytochemical report on its association with the kinetochore of progenitor cells [92], and despite a trend toward nuclear accumulation in CLPP-null MEFs (Figure S8). Possible explanations for these discrepancies include the technical differences, with mass spectrometry procedures fragmenting DNA by sonification while the fractionation protocol used DNA digestion by benzoase; immunocytochemical cross-reactivity with kinetochore components is a frequent artifact. Instead, the PEO1 immunoblot suggested altered isoform processing with less Twinkle (77 kDa, 0.4-fold, *p* = 0.055) but apparently more Twinky (66 kDa). Twinky was described in humans as unable to associate with the D-loop of mtDNA due to its loss of the C-terminal homo-multimerization domain [93]. PEO1 and HARS2 were the only PRLTS proteins with significant accumulation in mass spectrometry already at P17. HARS2, as the protein responsible for PRLTS2, showed not only a significant 1.5-fold mitochondrial accumulation (*p* = 0.0434), but also a completely novel redistribution to the nucleus even in the WT samples (Figure 5c). Although the presence of this mitochondrial PRLTS protein in the nucleus may contribute to the meiotic impairment, the mechanism remains unclear since the physiological effects of nuclear HARS2 are currently unknown. RMND1, responsible for PRLTS with renal involvement, was investigated although it did not appear dysregulated in the proteome survey, given that its first description in yeast and its name imply a role in meiosis. It exhibited accumulation within mitochondria in the immunoblots (2.7-fold, *p* < 0.0001), but no re-localization to the nuclear department.

## 4. Discussion

### 4.1. Exceptional Diplotene Arrest Phenotype in CLPP-Null Mice

Dysfunctional mitochondria have been connected to defective spermatogenesis in several mouse models. Male infertility with defects already observed in pachytene of prophase-I is a feature of mito-mice that inherit a pathogenic 4696-bp deletion in mtDNA [94], and still (aberrant) spermatids are formed in this mutant. Additionally, in mutator mice (a mutation of the mtDNA Polymerase Gamma (PolG)) [95], and in mice with deficiency of MTEF4 (mitochondrial translation control factor) [96], spermatids are still formed, albeit aberrant. Even in mice that lack two mitofusins (MFN1 and 2), where spermatogenesis is severely affected, already in early meiosis, some spermatids are formed [97]. In contrast, in Prohibitin (Prohibitin is a component of the inner mitochondrial membrane) (PHB)-deficient germ cells, there is a complete meiotic arrest at pachytene with incomplete chromosome pairing and meiotic DSB repair [98]. The complete diplotene arrest observed in CLPP-null mice is thus unique in its phenotype compared to other mitochondrial deficiencies, and also rather exceptional in comparison to arrest phenotypes in other mouse infertility models. Since meiosis arrests completely, and no post-meiotic cells are present, the composition of testes from adult mice is very different from controls (no spermatids, relative enrichment of all other earlier stages). However, at P17 and P21, the testes of CLPP-null mice and controls are still similar. Therefore, any gene or protein expression changes that are already apparent at this age can be attributed to actual dysregulation, and therefore we focused our attention on these.

It is known that DNA damage repair, chromosome synapsis/desynapsis, and spindle-dependent polarization are monitored by different surveillance factors, which can trigger apoptosis at specific checkpoints during spermatogenesis in mice. A recombination checkpoint during early pachytene, a DNA damage checkpoint during late pachytene, and a spindle checkpoint acting at metaphase-I have been characterized in detail [99,100]. Our observation in CLPP-null testes that chromosomes manage to complete synapsis and meiotic DSB repair correctly, but undergo precocious desynapsis, with MLH1 foci visible in diplotene-like nuclei, and re-accumulated γH2AX on desynapsed chromatin, represents a rare phenomenon with apoptosis during diplotene or diakinesis, but before entry into M-phase-I. This type of male meiotic arrest has recently been suggested to be a manifestation of a novel meiotic checkpoint in mice with the absence of IGSF11 [101]. Still, similar meiotic arrests between pachytene and before entry into M-phase-I were reported previously in mice with the absence of HSPA2 [54,55,102], HSPBP1 [103], EIF4G3 [104], EMI2 [105], SKP1 [106], CCNA1 [107,108], ZFP541 [109], RBMXL2 [110], and recently TCFL5 [111]. These mouse models can be grossly divided into two categories, the first involves mouse models for which it is clear that there is some interference with the basic machinery that actually mediates the transition from prophase-I into metaphase-I, involving well-known cyclin-dependent kinase CDK1 and its partner cyclin B, and this category includes (conditional) HSPA2-, HSPBP1-, EIF4G3-, EMI2-, SKP1-, and CCNA1-deficient mouse models. For the second category, the mechanisms by which the “diplotene arrest” occurs appear to be more multi-factorial, and/or may actually involve some type of checkpoint activation, since for IGSF11 (adhesion molecule), ZFP541 (Zn-finger protein), and RBMXL2 (RNA binding protein) deficiencies, there were reports of misregulation of heterochromatin, gene expression (in association with late DSB formation), and cryptic splice site poisoning, respectively [101,109,110,112,113]. For transcription factor TCFL5, the regulation of the meiotic transcriptome network, including the induction of piRNAs, was recently described [114,115]. The protein is expressed from early pachytene onward, reaching its maximal level in late diplotene, but is lost upon entry into meiotic M-phase [111,116]. In CLPP-null mice, expression is not detected at P17 in the mass-spectrometry data, and reduced to 56% and 45% at P21 and P27, respectively. This is consistent with the loss of late diplotene spermatocytes from the testes of CLPP-null mice, although we cannot exclude that the loss of this regulatory factor also contributes to the failure to complete diplotene.

In our CLPP-null mouse model, structural components that mediate the entry into M-phase may also be lacking since the mass spectrometry quantification of CLPP-null testis proteomes found the exceptional depletion of HSPA2 (13% at P17, 31% at P21, and 14% at P27), and its interactor [117] SHCBP1L (reductions to 0.4%, 13%, and 6%, respectively). The latter is the third most strongly downregulated protein at P17. HSPA2 has been directly connected with the transition from prophase-I into metaphase-I through its function as a molecular chaperone for CDK1 (CDC2) [102], but the HSPA2-interactor SHCBP1L has been characterized in less detail and the lack of this protein results in a milder phenotype, with reduced sperm count and aberrant meiotic metaphase morphology [117].

A possible link between the failure to progress beyond diplotene and mitochondrial dysfunction comes from the aberrant accumulation of the mitochondrial protein AURKAIP1 in the cytosol and/or nucleus of germ cells in CLPP-null testes. AURKAIP1 was identified on the one hand as a mitoribosomal component (also known as MRPS38), but on the other hand as a negative regulator of Aurora Kinase A, which localizes at centrosomes and is a key coordinator of anaphase [118,119,120,121,122]. AURKA deletion in spermatocytes leads to increased spermatocyte apoptosis [67]. A prominent phosphorylation target of Aurora Kinase A is Astrin (gene symbol *Spag5*). Protein levels of SPAG5 and its interactor KNSTRN exhibited the moderate downregulation in CLPP-null P21 and P27 testes, but these proteins are expressed in both spermatocytes and spermatids, so at least part of this may be due to the loss of cells expressing the proteins.

Finally, in the context of a possible checkpoint that could be activated in diplotene, we should take the accumulation of γH2AX on the prematurely desynapsing chromosomes into account. Although these do not seem to recruit homologous recombination factors, as indicated by the lack of RPA accumulation, and in contrast to what was observed at novel DSB sites in the IGSF11-null model, DNA damage-detecting proteins may be involved in activating a diplotene checkpoint and subsequent apoptosis in both instances of diplotene arrest. The γH2AX signal associated with chromosome regions that are prematurely desynapsing in CLPP-null diplotene spermatocytes may represent the defective processing of persistent very late meiotic DSB repair intermediates (post RAD51 and/or RPA accumulation) that may somehow trigger a re-accumulation of γH2AX, and perhaps be repaired through proteins acting in the re-activated (from mid-pachytene onward) non-homologous end-joining DSB repair pathway [123]. Alternatively, γH2AX accumulation may result from DNA damage caused by lack of proper chromatin structure re-organization such as chromatid individualization. Additionally, the trend toward increased H3K9 acetylation in CLPP-null testes, as well as the immunoblot evidence of H3 cleavage, point to a selective early affection of histone-3 regulation. Finally, there may be a relation between the specific observation of γH2AX accumulation along the desynapsing regions, and the re-appearance of HORMAD1 along these axes. In early pachytene, the accumulation of HORMAD1 and HORMAD2 along the unsynapsed axes of the XY pair is important for the subsequent intense accumulation of γH2AX immunosignal on the XY body, while the reappearance of HORMAD1 in diplotene does not have such an effect [27,124]. If desynapsis occurs too early, it cannot be excluded that the (otherwise normal) HORMAD1 re-appearance triggers γH2AX formation in the absence of (novel or persisting) DNA damage. Still, γH2AX accumulation-mediated DNA damage-signaling in CLPP-null diplotene nuclei cannot fully explain the complete arrest phenotype since not all diplotenes display this phenotype.

Previous reports suggested that the H3 cleavage in the nucleus and the early proteome deficit of COXFA4L3/NMES1 in the mitochondria could both be consequences of the innate immunity activation [52,82] that typically occurs in CLPP-null cells [16,17]. These findings provide another possible link between (DNA damage in) meiosis-I, inflammation, methylation anomalies, and mitochondria.

Scrutinizing the proteome for additional clues to understand the retrograde signaling from mitochondria to nuclei, the early dysregulation of several COX-associated factors was conspicuous. Within mitochondria overall, CLPP absence is known to have a prominent impact on the inner-membrane COX complex activity, the rate-limiting step of respiration, as previously reported [1], and on lactate metabolism, while metabolic functions such as acetylation or methylation in this study showed no gross anomaly. COX function has to be adjusted to testis-specific needs by the isoforms COX6B2 and COX7B2, and it is adapted to antiviral stress by the subunit COXFA4L3. These proteins showed outstanding stable downregulations in testis already at P17. Furthermore, the COX activity repressor PTCD1 exhibited early and strong upregulation, as shown in Figure 3 and Figure 5. However, bioenergetic and metabolic deficits so far mostly impair spermatogenesis only to a level known as asthenozoospermia, as indicated above [4].

An early consistent and massive anomaly of CLPP-null testis is the accumulation of mitochondrial RNA/DNA-associated proteins. The analysis of testis subcellular fractions by immunoblots showed at least HARS2 to spill from mitochondria and relocalize to nuclei, and we cannot exclude that such proteins have an impact on genome organization in the spermatocytes.

### 4.2. Premature Desynapsis in CLPP-Null Mice

The first phenotypic aberration in CLPP-null spermatocytes is the premature desynapsis in association with MLH1 foci persistence in diplotene nuclei. None of the known components of the synaptonemal complex were significantly downregulated at P17, precluding a simple explanation for this observation. MLH1 normally accumulates specifically during mid-to-late pachytene and is no longer or scarcely observed in diplotene spermatocytes [23,125,126,127,128,129,130,131,132]. Only two papers describe frequent MLH1 foci formation in wild-type diplotene spermatocytes [133,134]. Previously, it was reported that the experimental premature induction of diplotene using okadaic acid is also associated with MLH1 foci remaining present along the desynapsing synaptonemal complex [125,126]. Interestingly, only in two other mouse mutant models has the aberrant persistence of MLH1 foci in diplotene been reported and both mutant genes encode (components of) ubiquitin ligases: SKP1 [106] and CUL4A [135]. *Skp1*-KO mice display a complete arrest in diplotene while in *Cul4a*-KO mice, the arrest is incomplete, with cell apoptosis occurring at pachytene and/or diplotene and some formation of aberrant spermatids. In conditional *Skp1*-KO mice, premature desynapsis and persistence of MLH1, in conjunction with a failure to enter M-phase was observed [106], which is more similar to our phenotype. However, in the SKP1-deficient spermatocytes, the aberrant accumulation of HORMAD proteins and DSB-repair foci marked by RPA were observed [106], and these patterns were normal in the CLPP-null mice, indicating a different mechanism. Although HSPA2 has been proposed as a factor required for desynapsis [54], possibly via the stimulation of CDK1 activity [102], its downregulation is difficult to fit with our observations, where reduced HSPA2, and possibly impaired CDK1 activity would be associated with premature desynapsis. Finally, PLK1 has been shown to also be required for proper SC disassembly [136], but it is approximately 1.5-fold downregulated in P21 and P27 CLPP-null proteomes. Therefore, this also does not provide a mechanistic link between the premature desynapsis and the aberrant proteomes that are caused by the dysfunctional mitochondria. Further studies will be required to understand the connection between pathogenesis and other robustly downregulated but poorly characterized testis-enriched proteins such as the transcription factors DMRTC2 and HSF5, the kinase PBK, and the RNA-binding protein PABPC6.

### 4.3. Early and Robust Depletion of Proteins Associated with Ciliopathies

Many of the mouse models for mitochondrial dysfunction report aberrations of development of the flagellum of the sperm, which is to be expected due to the tight connection between the mitochondrial midpiece and the flagellum. However, in our CLPP-null mouse model, no spermatids are formed and the downregulation of components of the spermatid flagellum would most likely be mostly attributable to the loss of these cells. Still, at P17, when spermatids are expected to be extremely scarce, we could detect several cilium components in WT, and their strong and significant reduction in the mutant; DNALI1, DNAAF1, CCDC65, CCDC39, and LRRC34 were all downregulated (17–31% of wild-type at P17). Indeed, as described in the results section, in silico data point to the expression of these proteins in spermatocytes as well as spermatids, so these proteins may in fact be downregulated in spermatocytes. If and how cilia could be important for spermatocytes is at present fully unclear, but it is important to note that a very recent functional analysis of cilia components in zebrafish revealed the importance of cilia in the regulation of meiotic recombination at least in this species [137,138], and a cilium has been detected in part of the early zygotene spermatocytes in mice. Therefore, we cannot simply dismiss the possibility that ciliary protein downregulation may be a functionally connected to the observed meiotic defects, and that this may also affect meiotic prophase progression. Ciliary dysfunction had also been linked to mitochondrial dysfunction in previous reports [139,140,141,142], in good correlation the observations in our CLPP-null mouse model.

### 4.4. The Innate Immune Response in CLPP-Null Spermatocytes

ELISA evidence of increased cGAMP levels in the testis tissue confirmed that the STING-cGAS antiviral signaling pathway is activated, and an extrusion of mtDNA to the cytosolic compartment as a specific trigger was demonstrated by qPCR. Mitochondrial, bacterial, and viral nucleotide chains are abnormally hypomethylated in comparison with eukaryotic DNA/RNA and are thus recognized as potentially threatening by cytosolic surveillance factors. Thus, a chronic inflammatory process is present and likely contributes to the progressive atrophy of CLPP-null testis during ageing. However, the azoospermia was not mitigated when downstream immune signals were blocked by the genetic deletion of STING and IFNAR, so inflammation is probably not the primary cause for the meiosis-I arrest.

### 4.5. Abnormal Accumulation of Mitochondrial Proteins in Cytosol and Nucleus in Relation to the Meiotic Phenotype

An early and consistent proteome dysregulation is the progressive extra-mitochondrial decrease in molecular chaperones, with HSPA2 prominently among them. The transcription of all chaperones depends on the HSF family of transcription factors [143], so the significant downregulation of the poorly characterized HSF5 in the testis proteome may underlie this observation. A potential mitochondrial trigger of this nuclear anomaly may be the early accumulation of mitochondrial DNAJA3, which is redistributed to the nucleus where it influences STAT transcription factors [17]. It is conceivable that an excess of chaperones in mitochondria may retrogradely signal to the nucleus to suppress transcriptional biosynthesis of additional chaperones, as well as in the cytosol. This scenario provides a third connection between mitochondria and nuclear events, via deoxyribonucleoprotein complex disassembly.

## 5. Conclusions

Here, we have identified that a CLPP-null testis shows complete meiotic arrest in diplotene, an exceptional phenotype both for mitochondrial dysfunctions as well as for male meiotic dysfunction in general. This diplotene arrest is clinically relevant since premetaphase arrest (with normal progression of chromosome pairing and repair) was observed in 15% of azoospermic patients with spermatogenic defects (Johnson score 3–6) [144]. We also report the first developmental CLPP-null testis proteome profiles. The absence of mitochondrial CLPP as the PRLTS3 disease protein was observed to trigger the accumulation of several other PRLTS-responsible factors, namely ERAL1 as previously shown, but also HARS2 and PEO1 as novel insights.

Mechanistically, we identified the downregulation of factors that may explain part of the meiotic phenotype: HSPA2, SHCBP1L, DMRTC2, and HSF5. These molecules may be connected to the premature desynapsis and failure to enter meiotic M-phase-I. Additional findings of selective H3 affection, cytosolic extrusion of mtDNA, and increased cGAMP levels suggested a chronic activation of innate immunity due to methylation anomalies and mitochondrial toxicity as an underlying mechanism, but a block of inflammation by the ablation of STING and IFNAR failed to alleviate testis pathology. Interestingly, activation of cGAS has recently been linked to an infection sensor function of HORMA domain proteins in a bacterial immune system [145], and therefore it is tempting to suggest that CLPP deficiency could somehow induce a functional connection between meiotic HORMAD proteins and nuclear cGAS activities, contributing to cellular demise. An additional connection from mitochondria to nuclear events was identified in view of the accumulation of the mitoribosomal subunit MRPS38 (also known as AURKAIP1), which has the capacity to bind Aurora kinase A, and could therefore contribute to meiosis-I pathology. Finally, we noted the unexpected early downregulation (P17) of several proteins involved in ciliary functions, known to also be expressed in spermatocytes, and hinting at a possible function of cilia in meiotic prophase progression. It could now be highly informative to analyze CLPP-null oocytes prenatally as well as postnatally to investigate whether the diplotene arrest that is observed in CLPP-null males is somehow recapitulated in females, where during embryonic oogenesis all oocytes arrest in diplotene, to continue only just before ovulation after puberty has commenced. Many meiotic processes show sexual dimorphism in their requirements, but the infertility of both sexes in the CLPP-null mouse model does not yet reveal whether this could be the case. Overall, the findings identify various potential mechanisms of retrograde signaling from mitochondria to nucleus, which may conspire to abort CLPP-null spermatogenesis before metaphase-I.

## Data Availability

Testis LC–MS proteomics data have been deposited in the ProteomeXchange Consortium via the PRIDE [146] partner repository with the PXD033388.

## Supplementary Materials

The following are available online at https://www.mdpi.com/article/10.3390/cells12010052/s1. Figure S1: Additional meiotic analysis of CLPP-null spermatocytes. Figure S2: Additional examples of γH2AX immunostainings of pachytene and diplotene spermatocytes of CLPP-null mice, and quantification of the number of γH2AX flares. Figure S3: Additional examples of MLH1 immunostainings of pachytene and diplotene spermatocytes of CLPP-null mice. Figure S4: CLPP-null testis global proteome volcano plots at three ages. Figure S5: Immunohistochemistry of CLPP-null oviduct. Figure S6: Quantitative immunoblots to validate proteome profile. Figure S7: PCR quantification of mtDNA in subcellular fractions of 6–12-month-old testis tissue, ELISA quantification of testis homogenate cGAMP levels, immunoblot of cGAS in cytosolic and nuclear testis fractions, and H&E staining of double mutant mice with CLPP-null and absence of either STING or IFNAR. Figure S8: PEO1 isoforms in CLPP-null MEF fractions. Table S1. Mass-spectrometry profile of CLPP-null testis proteome at postnatal days P17, P21, and P27.

## Abbreviations

AAA+	ATPases associated with diverse cellular activities
ABC	Ammonium bicarbonate
ACN	Acetonitrile
ACRV1	Acrosomal Vesicle Protein 1
ACTB	Actin Beta
ADAM3	ADAM Metallopeptidase Domain 3A
AGC	Automatic gain control
ALKBH7	AlkB Homolog 7
AURKA	Aurora Kinase A
AURKAIP1	Aurora Kinase A Interacting Protein 1
AURKC	Aurora Kinase C
BCA	Bicinchoninic acid assay
BET	Bromodomain and extraterminal domain
BIRC5	Baculoviral IAP Repeat Containing 5
BRDT	Bromodomain testis associated
BSA	Bovine serum albumin
CDCA8	Cell Division Cycle Associated 8
CENPE	Centromere Protein E
CENPU	Centromere Protein U
cGAMP	Cyclic guanosine monophosphate–adenosine monophosphate
cGAS	Cyclic GMP-AMP Synthase
CLPP	Caseinolytic Mitochondrial Matrix Peptidase Proteolytic Subunit
CLPX	Caseinolytic Mitochondrial Matrix Peptidase Chaperone Subunit X
CPC	Chromosome passenger complex
CSNK1D	Casein Kinase 1 Delta
DAZL	Deleted In Azoospermia Like
DCP1A	Decapping MRNA 1A
DMC1	DNA Meiotic Recombinase 1
DMRTC2	Doublesex- and Mab-3-Rrelated transcription factor C2
DNA	Deoxyribonucleid acid
DNAAF	Dynein Axonemal Assembly Factor 1
DNAJA3	DnaJ Heat Shock Protein Family (Hsp40) Member A3
DNAJB13	DnaJ Heat Shock Protein Family (Hsp40) Member B13
DNAJB3	DnaJ Heat Shock Protein Family (Hsp40) Member B3
DNALI1	Dynein Axonemal Light Intermediate Chain 1
DSB	Double-strand break
ELISA	Enzyme-linked immunosorbent assay
EPPS	*N*-(2-Hydroxyethyl)piperazine-*N*′-(3-propanesulfonic acid)
ERAL1	Era Like 12S Mitochondrial RRNA Chaperone 1
ESPL1	Extra Spindle Pole Bodies Like 1, Separase
FA	Formic acid
FZR1	Fizzy And Cell Division Cycle 20 Related 1
GAPDH	Glyceraldehyde-3-Phosphate Dehydrogenase
GFM1	G Elongation Factor Mitochondrial 1
γH2AX	Gamma histone H2A
GRSF1	G-Rich RNA Sequence Binding Factor 1
GTP	Guanosine triphosphate
H	Hour
H3S10p	Histone 3 Serine 10 phosphorylation
HARS2	Histidyl-TRNA Synthetase 2, Mitochondrial
HCD	High-energy collision-induced dissociation
HILS1	H1.9 Linker Histone, Pseudogene
hnRNP	Heterogeneous Nuclear Ribonucleoprotein
HPLC	High-performance liquid chromatography
HR	Homologous recombination
HSF5	Heat Shock Transcription Factor 5
HSP60	Heat Shock Protein Family D (Hsp60) Member 1
HSPA1L	Heat Shock Protein Family A (Hsp70) Member 1 Like
ID	Inner diameter
IFNAR	Interferon (alpha and beta) receptor 1
INCENP	Inner Centromere Protein
kDA	kiloDalton
KO	Knockout
LARS2	Leucyl-TRNA Synthetase 2, Mitochondrial
LC–MS	Liquid chromatography–mass spectrometry
LDHC	Lactate Dehydrogenase C
LRPPRC	Leucine Rich Pentatricopeptide Repeat Containing
MAD2L2	Mitotic Arrest Deficient 2 Like 2
MAVS	Mitochondrial antiviral signaling protein
MS	Mass spectrometry
MEFs	Mouse embryonic fibroblasts
Min	Minute
MLH1	DNA Mismatch Repair Protein MutL Homolog 1
MLH3	MutL (*E. Coli*) Homolog 3
mRNA	Messenger RNA
MRPL43	Mitochondrial Ribosomal Protein L43
MS	Mass spectrometry
mtDNA	Mitochondrial DNA
mtRNA	Mitochondrial RNA
n.d.	Not detected
NANOS3	Nanos C2HC-Type Zinc Finger 3
NCAPG2	Non-SMC Condensin II Complex Subunit G2
NCE	Normalized collision energy
NLR	Nod-like receptor
NMES1	Normal mucosa of esophagus-specific gene 1
NOS	Non-obstructive azoospermia
PAS	Periodic acid-Schiff
PBS	Phosphate-buffered saline
PCGF6	Polycomb Group Ring Finger 6
PD	Proteome Discoverer software
PEO1	Progressive external ophthalmoplegia (=Twinkle)
PGK2	Phosphoglycerate Kinase 2
PLK1	Polo Like Kinase 1
POLDIP2	DNA Polymerase Delta Interacting Protein 2
PolG	Polymerase Gamma
PPP2R5C	Protein Phosphatase 2 Regulatory Subunit B’Gamma
PRLTS	Perrault syndrome
PRM3	Protamine 3
PRORP	Protein Only RNase P Catalytic Subunit
PSM	Peptide-spectrum matches
PTCD1	Pentatricopeptide Repeat Domain
PTTG1	PTTG1 Regulator Of Sister Chromatid Separation, Securin
qPCR	Quantitative polymerase chain reaction
RAD21L	RAD21 Cohesin Complex Component Like 1
REC8	REC8 Meiotic Recombination Protein
RF	Radio frequency
RHAU	RNA Helicase Associated With AU-Rich Element Protein
RIPA	Radioimmunoprecipitation assay buffer
RMND1	Required For Meiotic Nuclear Division 1 Homolog
RNA	Ribonucleic acid
RPA	Replication Protein A
Rpm	Revolutions per minute
RT-qPCR	Reverse transcriptase quantitative polymerase chain reaction
s	Second
SAC	Spindle assembly checkpoint complex
SC	Synaptonemal complex
SDS	Sodium dodecylsulfate
SEM	Standard error of the mean
SGOL2	Shugoshin 2
SHCBP1L	SHC Binding And Spindle Associated 1 Like
SMC1B	Structural Maintenance Of Chromosomes 1B
SMC3	Structural Maintenance Of Chromosomes 3
SPACA4	Sperm Acrosome Associated 4
SPAG4	Sperm Associated Antigen 4
ssDNA	Single-strand DNA
STAG1/2/3	Stromal Antigen 1/2/3
STAT1	Signal Transducer And Activator Of Transcription 1
STING	Stimulator Of Interferon Response CGAMP Interactor 1
STRA8	Stimulated By Retinoic Acid 8
STRING	Search tool for recurring instances of neighboring genes
SUN3	Sad1 and UNC84 domain containing 3
SYCE1L	Synaptonemal Complex Central Element Protein 1 Like
SYCP3	Synaptonemal Complex Protein 3
TBP	TATA-Box Binding Protein
TCEP	Tris(2-carboxyethyl)phosphin
TEX12	Testis Expressed 12
TFAM	Transcription Factor A, Mitochondrial
Th	Thomson (mass-to-charge ratio as mass spectrometry unit)
TID1S	Tumorous Imaginal Discs Protein Tid56 Homolog
TLR	Toll-like receptor
TMT	Tandem mass tag
TNRC6A	Trinucleotide Repeat Containing Adaptor 6A
TRIM33	Tripartite Motif Containing 33
tRNA	transfer RNA
tsHMG/TFAM	Testis-specific High-Mobility Group protein (TFAM testis isoform)
TSSK1B	Testis-Specific Serine Kinase 1B
TUNEL	Terminal deoxynucleotidyl transferase dUTP nick end labeling
VWA8	Von Willebrand Factor A Domain Containing 8
WT	Wild-type
XY body	Chromatin domain of the X and Y chromosomes
ZFE	Central animal facility
