# Supplementary figures and images for: CLPP Depletion Causes Diplotene Arrest; Underlying Testis Mitochondrial Dysfunction Occurs with Accumulation of Perrault Proteins ERAL1, PEO1, and HARS2
Source: Cells. 2022 Dec 22;12(1):52. doi: 10.3390/cells12010052 (PMC9818230; doi:10.3390/cells12010052)

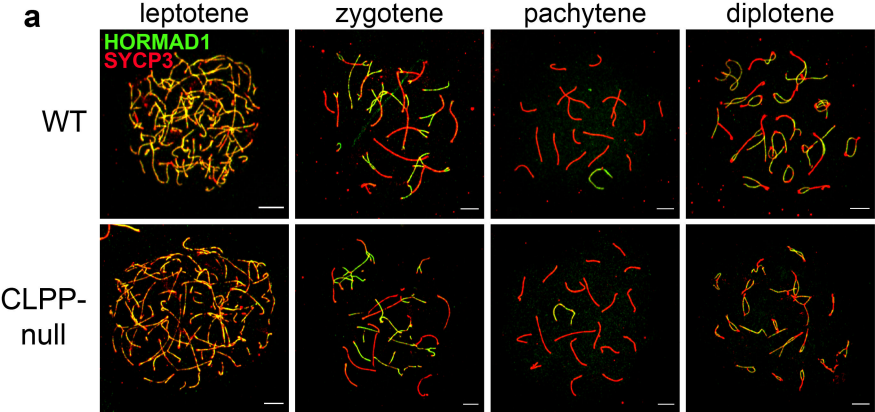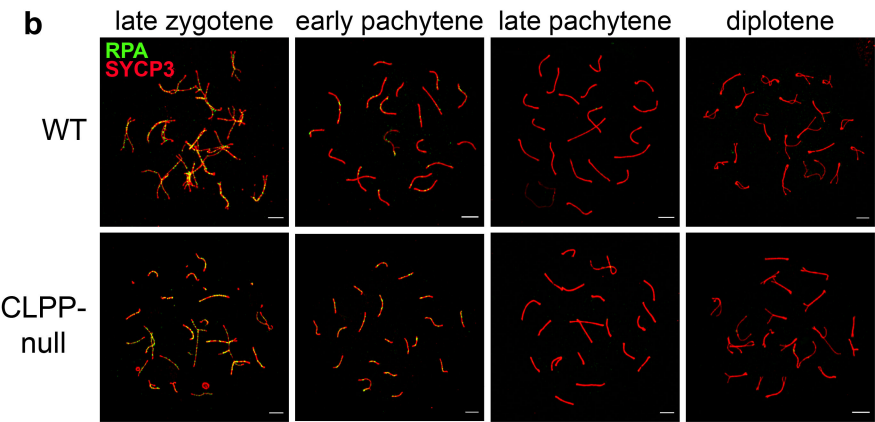

Supplement: Supplementary file 1 [file cells-12-00052-s001.zip › FigureS1-KeyAuburger.pdf]

**pachytene**

WT

CLPP-null

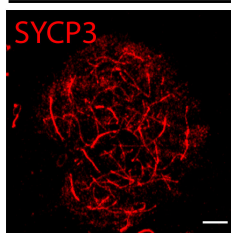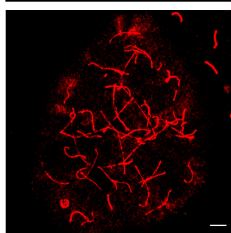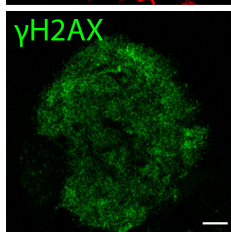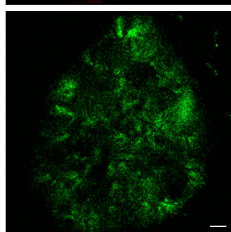

WT

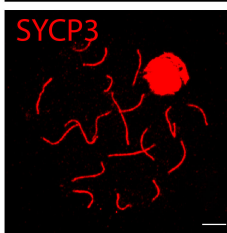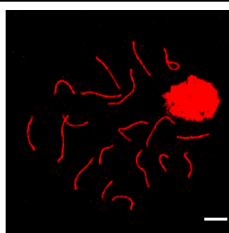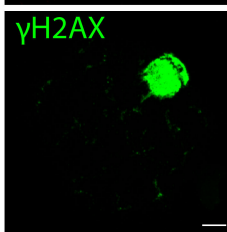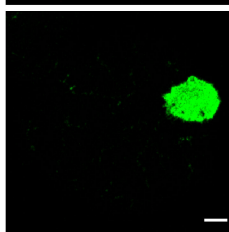

CLPP-null

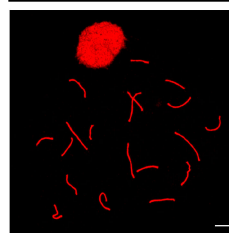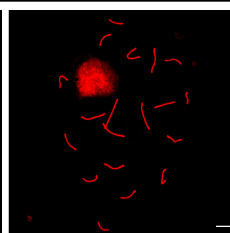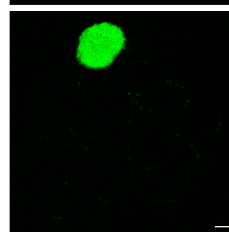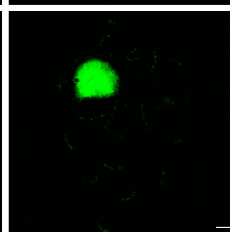

WT

**diplotene**

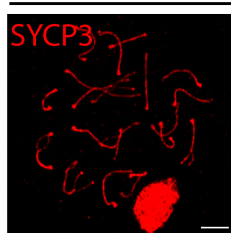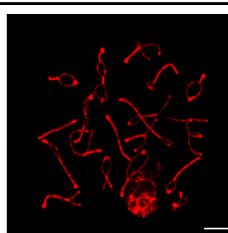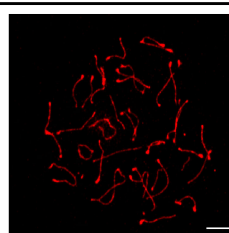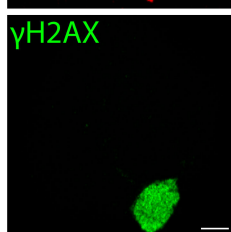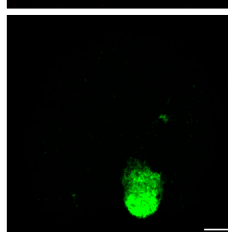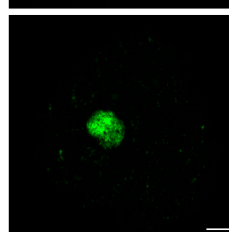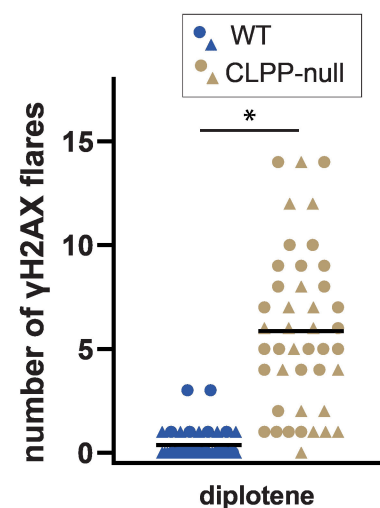

CLPP-null

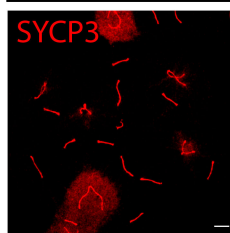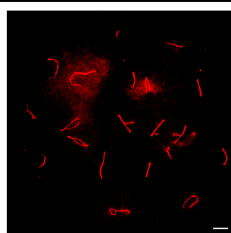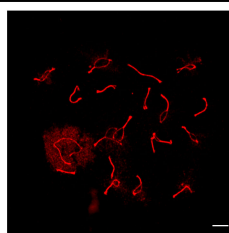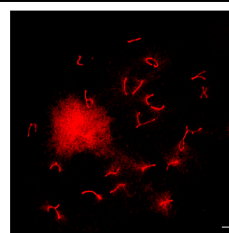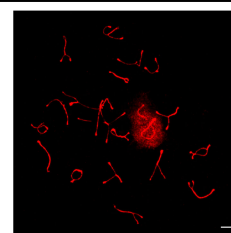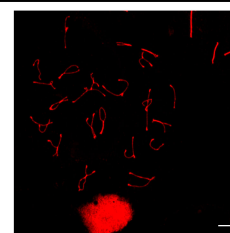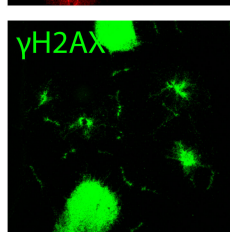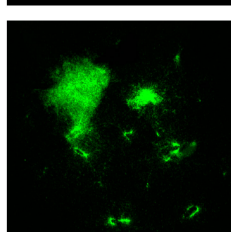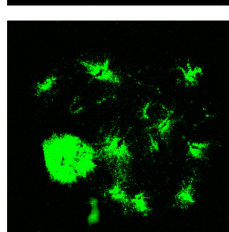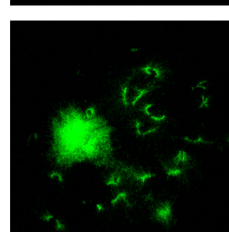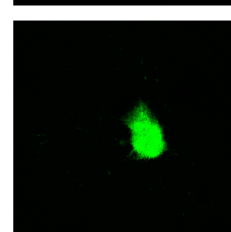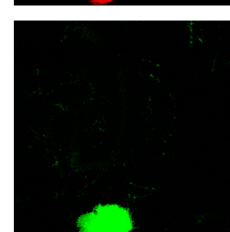

Supplement: Supplementary file 1 [file cells-12-00052-s001.zip › FigureS2-KeyAuburger.pdf]

## pachytene

WT

CLPP-null

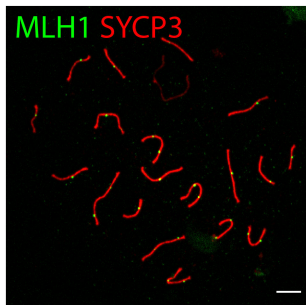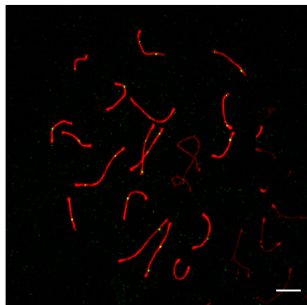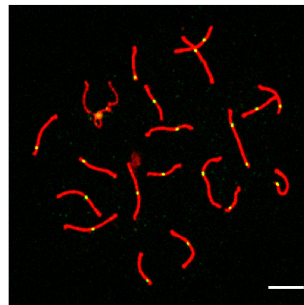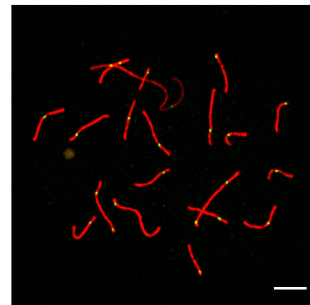

## diplotene

WT

CLPP-null

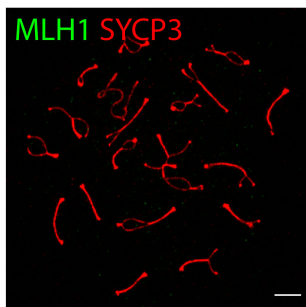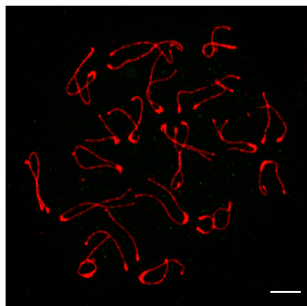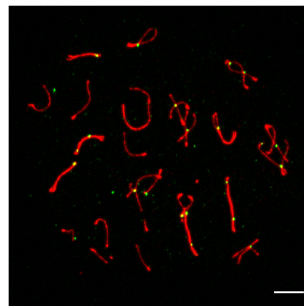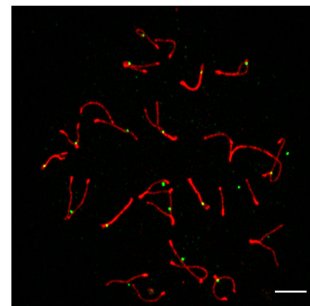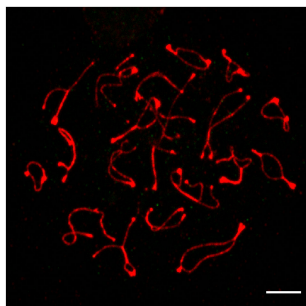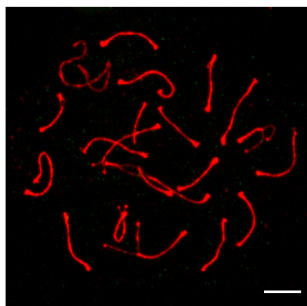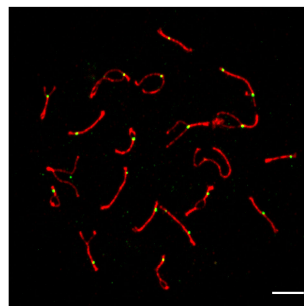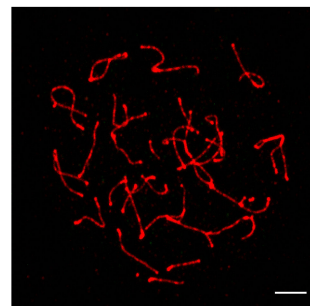

Supplement: Supplementary file 1 [file cells-12-00052-s001.zip › FigureS3-KeyAuburger.pdf]

## P17

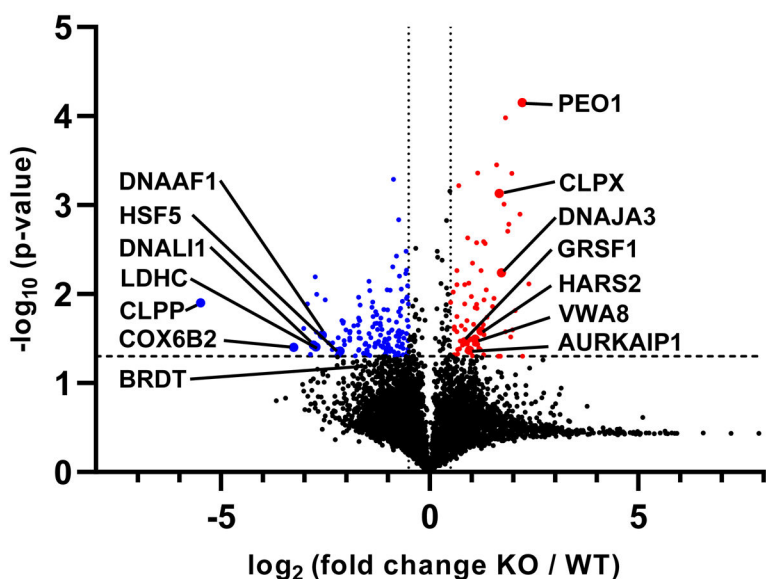

## P21

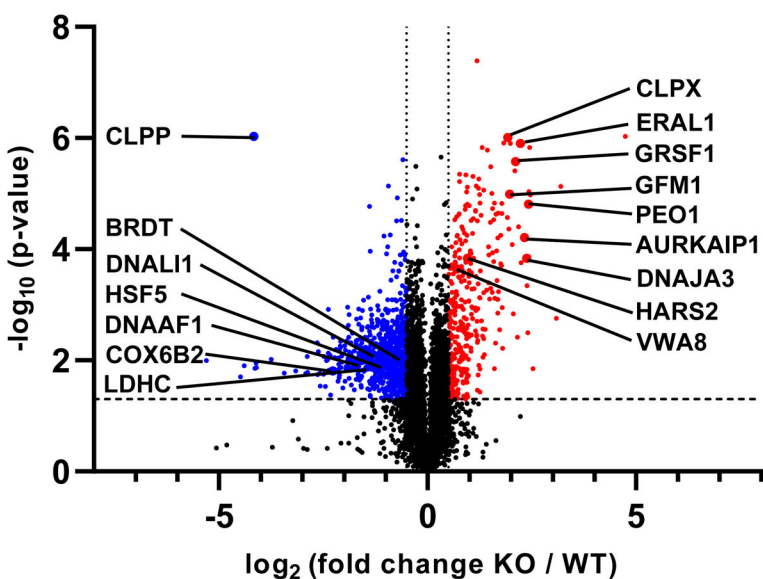

## P27

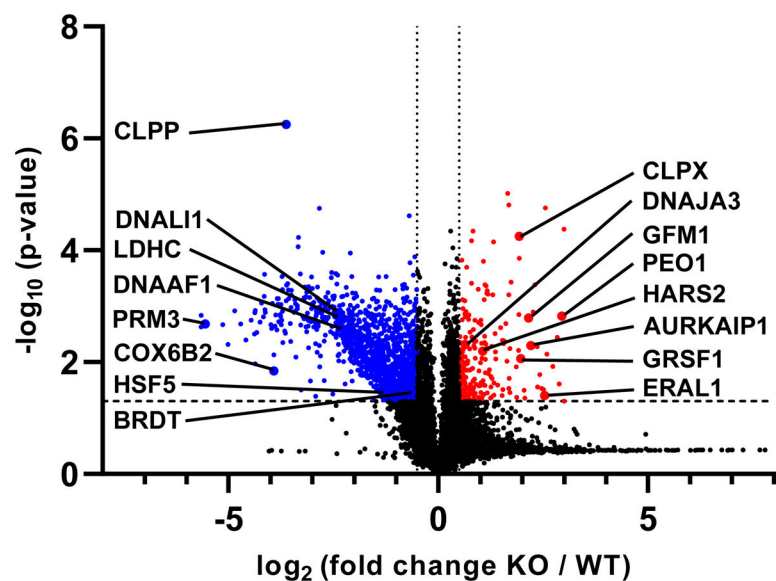

Supplement: Supplementary file 1 [file cells-12-00052-s001.zip › FigureS4-KeyAuburger.pdf]

**WT oviduct cilia DNAI1**

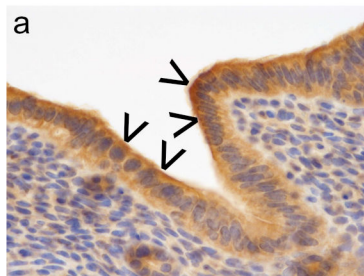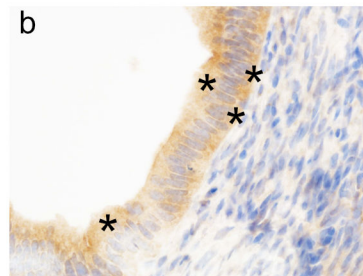

***Clpp*<sup>-/-</sup> oviduct cilia DNAI1**

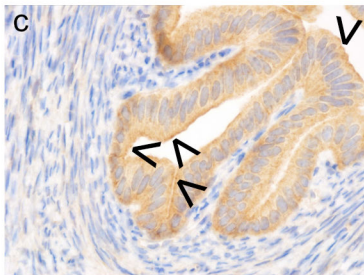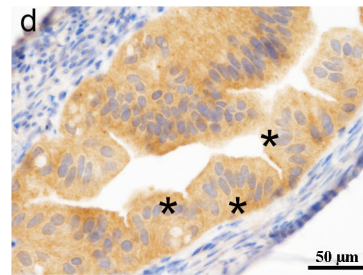

Supplement: Supplementary file 1 [file cells-12-00052-s001.zip › FigureS5-KeyAuburger.pdf]

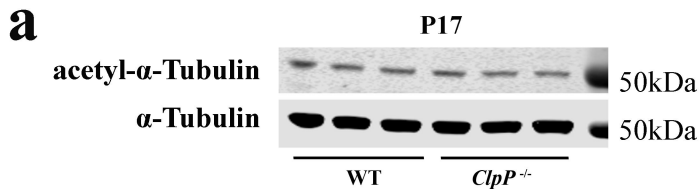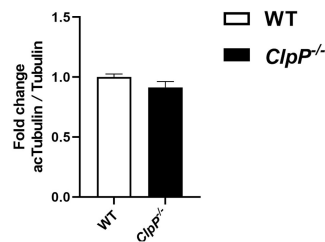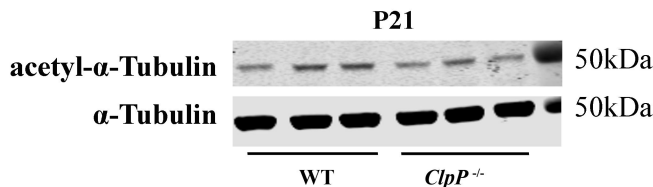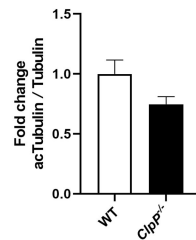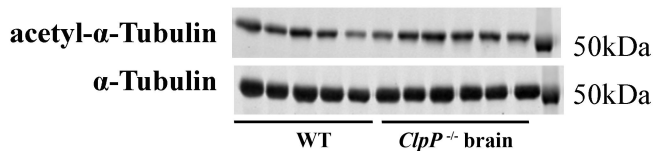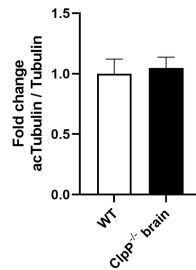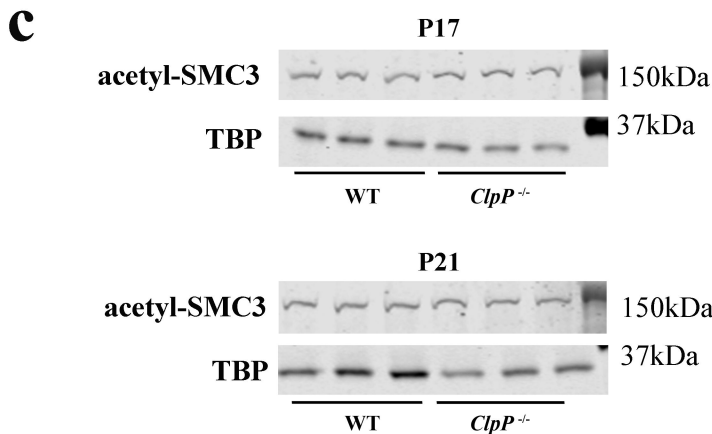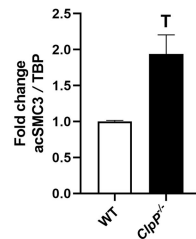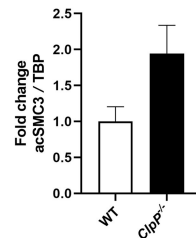

Supplement: Supplementary file 1 [file cells-12-00052-s001.zip › FigureS6-KeyAuburger.pdf]

**a**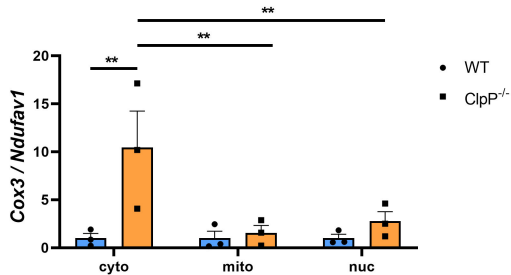**b**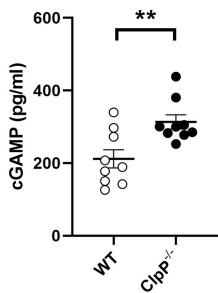**c**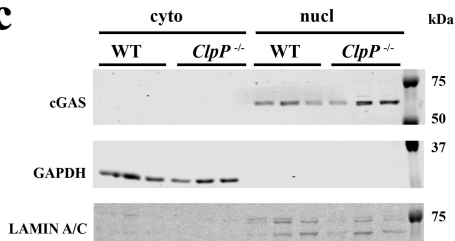**d**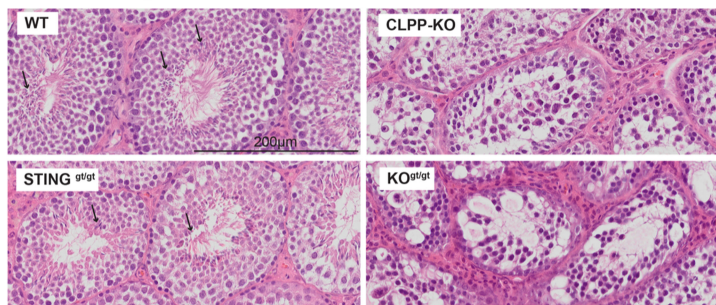**e**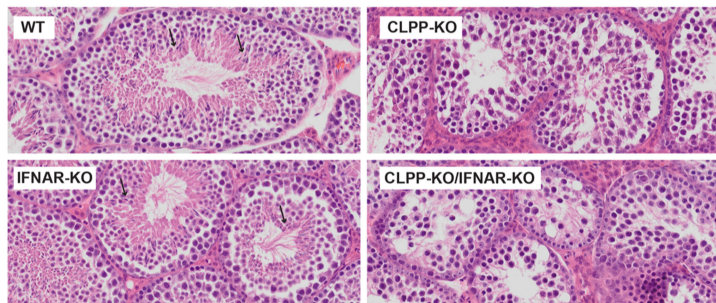

Supplement: Supplementary file 1 [file cells-12-00052-s001.zip › FigureS7-KeyAuburger.pdf]

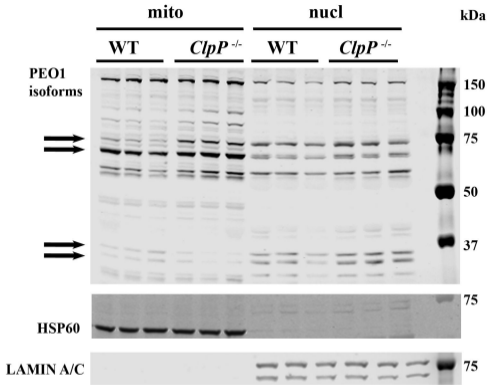

**mito 75kDa**

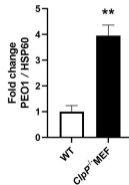

**nucl 75kDa**

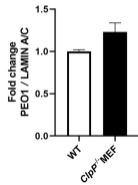

**mito 66kDa**

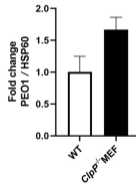

**nucl 66kDa**

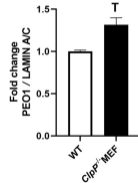

Supplement: Supplementary file 1 [file cells-12-00052-s001.zip › FigureS8-KeyAuburger.pdf]
